# Longitudinal development of the human white matter structural connectome and its association with brain transcriptomic and cellular architecture

**DOI:** 10.1038/s42003-023-05647-8

**Published:** 2023-12-12

**Authors:** Guozheng Feng, Rui Chen, Rui Zhao, Yuanyuan Li, Leilei Ma, Yanpei Wang, Weiwei Men, Jiahong Gao, Shuping Tan, Jian Cheng, Yong He, Shaozheng Qin, Qi Dong, Sha Tao, Ni Shu

**Affiliations:** 1 State Key Laboratory of Cognitive Neuroscience and Learning & IDG/McGovern Institute for Brain Research, Beijing Normal University, Beijing, China; 2 BABRI Centre, Beijing Normal University, Beijing, China; 3 Beijing Key Laboratory of Brain Imaging and Connectomics, Beijing Normal University, Beijing, China; 4 College of Life Sciences, Beijing Normal University, Beijing, China; 5 Beijing Key Laboratory of Gene Resource and Molecular Development, Beijing, China; 6 Center for MRI Research, Academy for Advanced Interdisciplinary Studies, Peking University, Beijing, China; 7 Beijing Huilongguan Hospital, Peking University Huilongguan Clinical Medical School, Beijing, China; 8 School of Computer Science and Engineering, Beihang University, Beijing, China

**Keywords:** Development of the nervous system, Brain, Network models

## Abstract

From childhood to adolescence, the spatiotemporal development pattern of the human brain white matter connectome and its underlying transcriptomic and cellular mechanisms remain largely unknown. With a longitudinal diffusion MRI cohort of 604 participants, we map the developmental trajectory of the white matter connectome from global to regional levels and identify that most brain network properties followed a linear developmental trajectory. Importantly, connectome-transcriptomic analysis reveals that the spatial development pattern of white matter connectome is potentially regulated by the transcriptomic architecture, with positively correlated genes involve in ion transport- and development-related pathways expressed in excitatory and inhibitory neurons, and negatively correlated genes enriches in synapse- and development-related pathways expressed in astrocytes, inhibitory neurons and microglia. Additionally, the macroscale developmental pattern is also associated with myelin content and thicknesses of specific laminas. These findings offer insights into the underlying genetics and neural mechanisms of macroscale white matter connectome development from childhood to adolescence.

## Abstract (web summary)

Joint connectomic and transcriptomic analyses of longitudinal dMRI data reveal the multiscale developmental pattern of the human white matter connectome from childhood to adolescence.

## Introduction

From childhood to adolescence, the neural circuitry of the human brain undergoes dramatic changes, which supports rapid behavior and cognitive development^1–4^. As the anatomical substrate of the neural circuitry, white matter (WM) shapes functional synchronization and undergoes extensive biophysical development, such as myelination, synaptic pruning, and increased axonal density^5,6^, which facilitates rapid neural signal communication between regions. Importantly, brain development exhibits heterogeneous patterns across different regions, and the primary sensorimotor cortex matures earlier than the higher-order association cortex^7^. Genes play an important role in regulating brain structural and functional development across age and regions^8–11^. Although previous studies have characterized the age-related trajectory of typical WM development^5^, the genetic and cellular mechanisms of WM development from a longitudinal perspective remain largely unknown.

As the brain is a complex system, network modeling and graph theory-based analyses have provided an important approach in investigating brain integration and segregation from a system level^12–15^. With diffusion MRI (dMRI) and tractography techniques, the whole-brain WM structural connectome can be delineated in vivo. This delineation captures the tangible fiber connections interconnecting distinct cerebral regions and unveils several nontrivial topological properties, such as small-worldness, modular structure, and rich-club organization^12,13,16^. With normal development, increased global and local efficiency, stable or decreased clustering, and the modularity of the WM structural connectome can be observed, typically indicating a WM network reconfiguration from being local to more distributed and integrated^17–21^. Our previous studies also revealed increased trade-off between the integration and segregation of the WM connectome with development, which may be the outcome of both the heterogeneous strengthening and the pruning of specific fibers^22–24^.

Longitudinal cohorts can be evaluated to characterize brain development trajectory more accurately than cross-sectional cohorts by disentangling within-person developmental change from between-person variation^5^. With a longitudinal cohort, a spatial refinement of WM connectivity between hub regions appears in late adolescence^25^. Another longitudinal study reported spatial distribution and topological differences with development across different edge types of the WM connectome^26^. However, there were hardly longitudinal studies with large samples on multiscale WM connectome development from global to regional/connectional levels, limiting insight into patterns and trends in multiscale WM connectome development.

Genes play important roles in regulating WM development. Typical twin studies have observed moderate to high heritability of specific WM tracts^27^, and genetic factors can mediate the relationship between WM microstructure and intelligence^28^. Furthermore, large-scale genome-wide association studies have found that the WM microstructure is regulated by hundreds of genes that are associated with brain neurodevelopment, cognitive functions and multiple brain disorders^10,29,30^, but these previous studies lack information of spatial variations on gene expression. The Allen Human Brain Atlas (AHBA, http://human.brain-map.org/) offered RNA expression levels of more than 20,000 genes taken from 3,702 spatially distinct brain tissue samples^31^, making it possible to bridge the gap between neuroimaging and transcriptomics^32^. With imaging transcriptomic analysis, genes whose expression pattern co-varying with brain imaging phenotypes can be identified and further enrichment analyses can be carried out to explore potentially functional pathways and cellular processes^11,33–35^. A recent functional network study investigated the association between modular variability with development and gene expression profiles, which identified the genes enriched for ion transport and nucleobase-containing compound transport^36^. Another study revealed that the transition of functional gradient during development is associated with the expression levels of calcium ion regulated exocytosis and synaptic transmission-related genes^37^. WM structural connectivity between brain regions has been shown to correlate with cortical gene expression using AHBA^38^. However, the transcriptomic architecture of WM connectome development remains largely unknown.

Developmental studies have demonstrated heterogeneous age-related increases in cortical myelination which may underlie the enhanced cognitive ability^35,39,40^. The age-related increases in cortical myelination accompanied by cortical shrinkage are maximized approximately at the internal layer of projection neurons^35^. Recently, a quantitative laminar atlas^41^ derived from a 3D histological atlas of the human brain at 20-micrometer isotropic resolution (BigBrain)^42^, provided high level of cytoarchitectonic detail to capture six cortical laminas formed by cellular division and differentiation. Thus, we attempted to establish a link between the developmental patterns of the macroscale WM connectome and microscale myelin content^43^ or cortical laminar thickness.

In the present study, we aimed to characterize the age-related longitudinal trajectory of the WM structure connectome from global, regional, and connectional levels, based on a large-sample cohort with 604 typically developing children from 6 to 13 years of age. To explore the potential genes regulating spatial patterns of WM connectivity development, we referred to the AHBA, and recognized the enrichment pathways and their cellular organizations based on connectome and transcriptome association analyses. Moreover, we examined whether the heterogeneous spatial development of the WM connectome can reflect the cytoarchitectural properties of cortical organization. Finally, different modeling methods and an independent development cohort were used to assess the reproducibility of our findings.

## Results

**Fig. 1:**
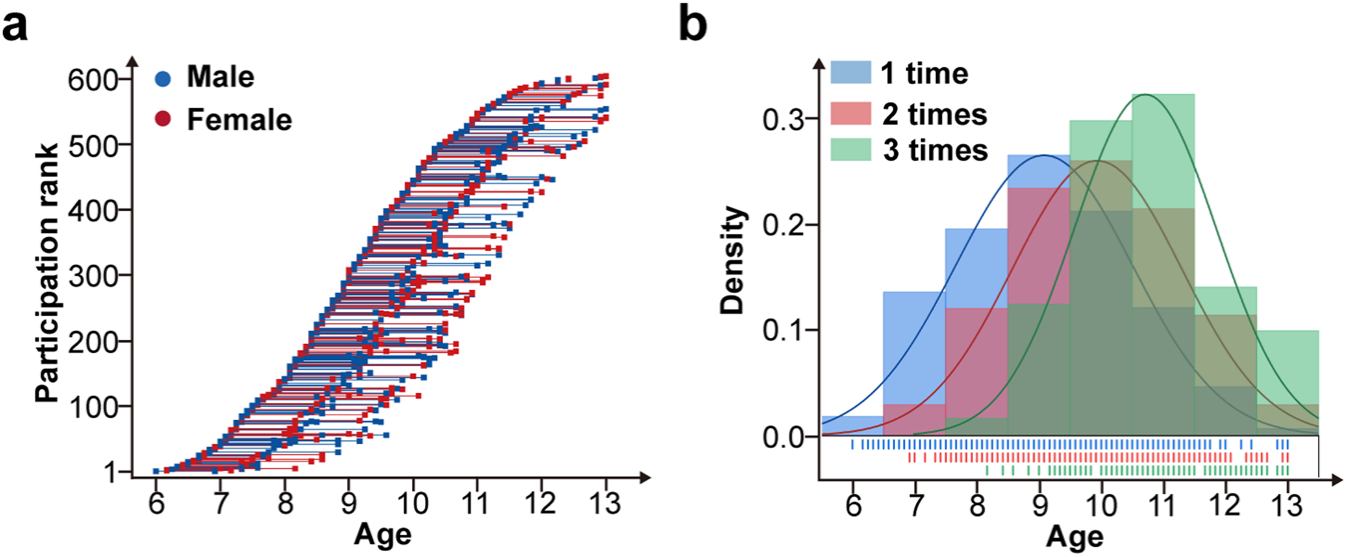
Age distributions of scans (*n* = 1033) in different sexes and different acquisition times. **a** Each point represents an individual scan, and the connecting lines indicate the interval between scans for each participant. **b** Age distribution of participants who completed different waves of scans. Of note, “1 time, 2 times, 3 times” represents the number of scans for each participant.

**Fig. 2:**
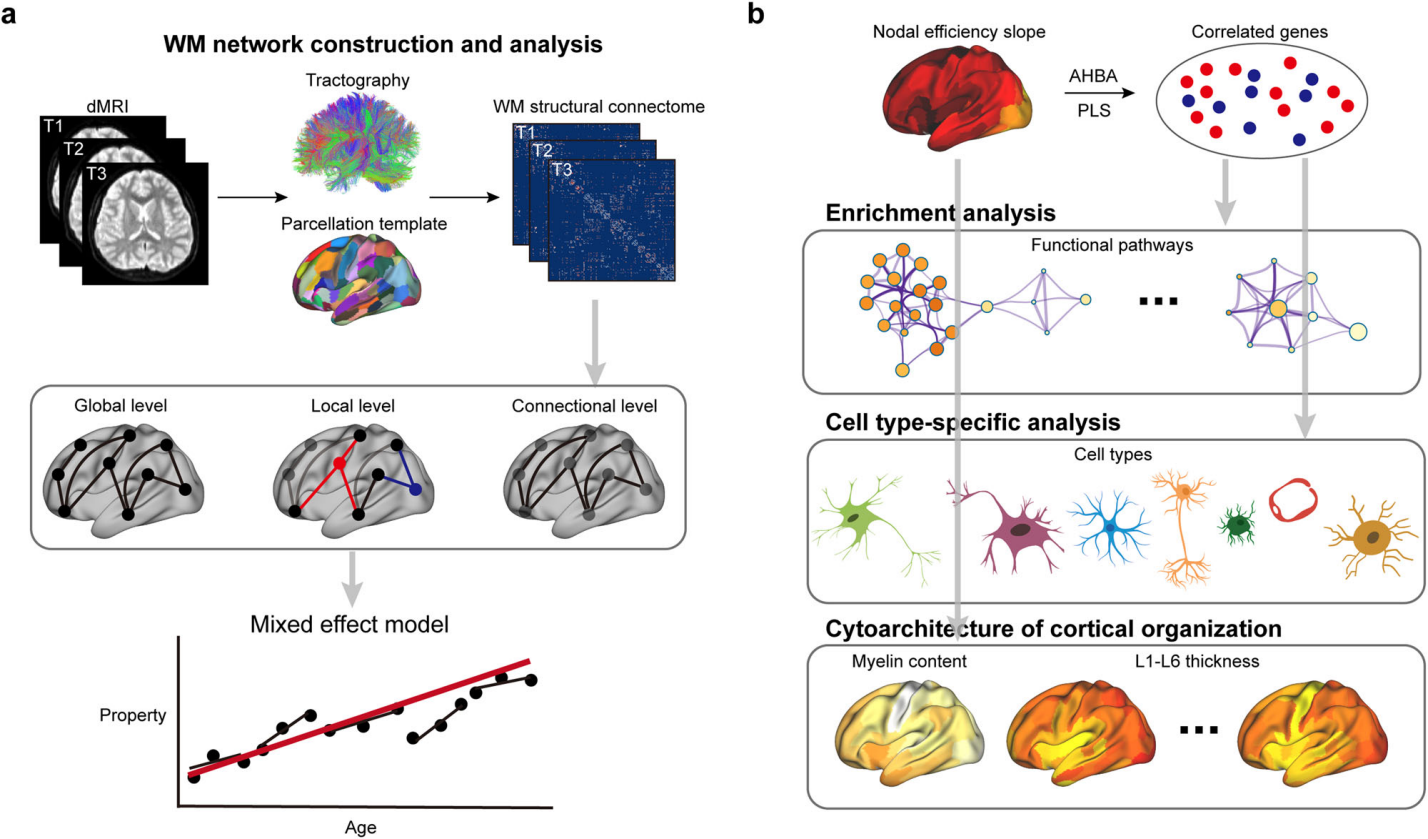
Overview of analysis workflow. **a** Based on WM network, the age-related longitudinal trajectories of global, regional, and connectional properties were analyzed using mixed effect model. **b** By correlating developmental slope of nodal efficiency and AHBA transcriptomic data, the significant genes were identified and were recognized the enrichment pathways and cell-type-specific expression. Meanwhile, the relationships between the developmental slope of nodal efficiency and the cytoarchitectural properties of cortical organization were identified.

In the present study, we used the 3-year longitudinal development data from the Children School Functions and Brain Development Project (CBD, Beijing Cohort)^44^, including 604 typically developing children from 6 to 13 years of age (339 males and 266 females) (Supplementary Table 1, Fig. 1). A total of 1033 scans taken at up to three time-point were used for following modeling and analysis. The overview of analysis workflow is shown in (Fig. 2). For validation analysis, we employed a cross-sectional development cohort from the Human Connectome Project in Development (HCP-D)^45,46^ (https://www.humanconnectome.org/study/hcp-lifespan-development), including 179 typically-developing children from 6 to 13 years of age (76 males and 103 females) who were unrelated to each other (Supplementary Table 2).

### Longitudinal developmental trajectory of the WM structural connectome

The network efficiency of the WM connectome reflects information integration, stands as a critical aspect of brain maturation, essential for information processing and cognitive functions^20^. The development of WM structural connectivity also promotes functional specialization^47–50^. For each participant, individual dMRI and T1 data were utilized to construct the brain WM structural connectome based on the Human Brainnetome Atlas with 246 brain regions (BNA246)^51^ template. Our study comprehensively delineates the development of the WM connectome in children aged 6 to 13 years from global, nodal, and connectional perspectives. We measured global integration and local segregation properties, including global efficiency, local efficiency, network strength, shortest path length, and clustering coefficient in relation to age. To understand the contribution of individual nodes (brain regions) to information transmission within the WM network, changes in nodal efficiency, nodal local efficiency, and nodal degree centrality were evaluated with age. Additionally, age-related changes were analyzed at the connection level, encompassing rich-club, feeder, and local edges; within-module and between-module edges; and long-range and short-range edges. For a detailed description of network properties, please refer to the Methods section. For each property, both linear and quadratic models were estimated by a mixed effect model^52^ to characterize the intrinsic longitudinal relationship between brain network properties and age. By comparing the Akaike information criterion^53^ of the linear and quadratic models, we found that most brain network properties followed a linear developmental trajectory over the age range of 6 to 13 years.

**Fig. 3:**
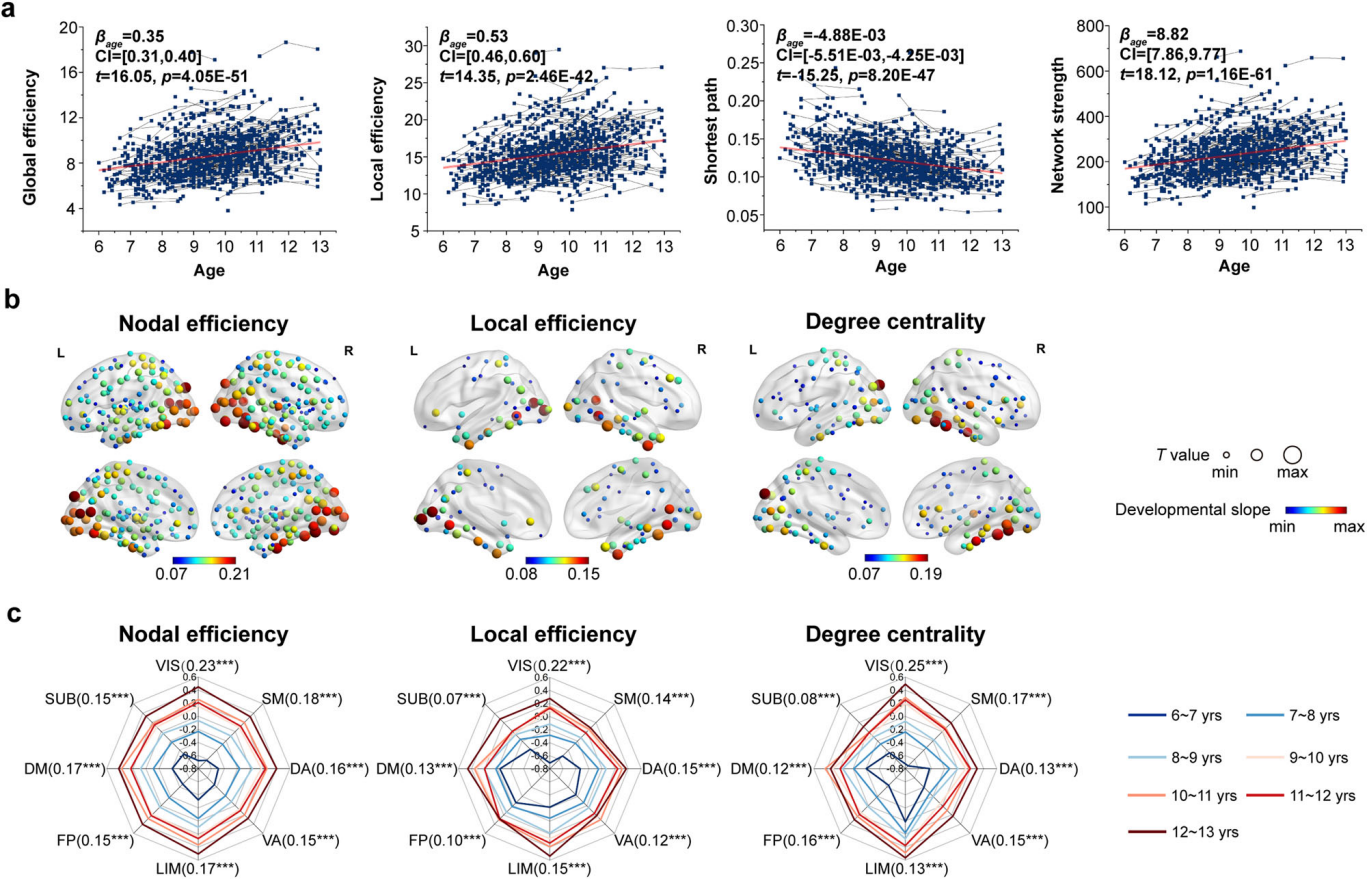
longitudinal changes in network organization properties during childhood and adolescence. **a** Age effect on mainly global network properties (*n* = 1033 scans). **b** Spatial patterns with significant development (*p* < 0.05, Bonferroni correction, *n* = 1033 scans) in various nodal properties. The size of the circle was proportional to the statistical *t* value, and its color indicated the developmental slope (standard effect value ${\beta }_{{age}}$), with red for large changes and blue for small changes. **c** Developmental slope of the functional subnetwork (*n* = 1033 scans). In each radar chart, a line with a different color corresponded to an age subgroup and values for each subnetwork were the average of properties over all scans in that subgroup. Of note, ***p < 0.001; n.s.*p* > 0.05, Bonferroni correction.

At the global level, we observed that the global efficiency (${\beta }_{{age}}$ = 0.35, CI = [0.31,0.40], *t* = 16.05, *p* = 4.05E-51), local efficiency (${\beta }_{{age}}$ = 0.53, CI = [0.46,0.60], *t* = 14.35, *p* = 2.46E-42) and network strength (${\beta }_{{age}}$ = 8.82, CI = [7.86,9.77], *t* = 18.12, *p* = 1.16E-61) of the whole-brain WM network linearly increased with age, and the shortest path length (${\beta }_{{age}}$ = −4.88E-03, CI = [−5.51E-03,−4.25E-03], *t* = −15.25, *p* = 8.20E-47) significantly decreased with age, as shown in Fig. 3a. The clustering coefficient (${\beta }_{{age}}$ = −1.23E-05, CI = [−9.08E-05,1.16E-05], *t* = 0.23, *p* = 0.81, Supplementary Fig. 1a) showed no significant changes with age. For small-world properties, $\gamma$ (${\beta }_{{age}}$ = −6.79E-02, CI = [−9.75E-02,−3.84E-02], *t* = −4.55, *p* = 6.05E-06) and $\sigma$ (${\beta }_{{age}}$ = −5.55E-02, CI = [−8.11E-02,−2.99E-02], *t* = −4.27, *p* = 2.16E-05) significantly decreased with age, and $\lambda$ (${\beta }_{{age}}$ = −1.14E-03, CI = [−3.21E-03,9.31E-04], *t* = −1.08, *p* = 0.28) remained stable with age, as shown in Supplementary Fig. 1b–d. The effects of sex and age-by-sex interaction were nonsignificant for all global network properties (*p* > 0.05, Bonferroni corrected).

At the regional level, we calculated the nodal efficiency, nodal local efficiency and nodal degree centrality for each brain region. For nodal efficiency, 229 regions exhibited a linear increase with age (*p* < 0.05, Bonferroni corrected), with various development slopes (${\beta }_{{age}}\in [0.07,0.21]$, Fig. 3b), which were distributed across most regions of the brain. For nodal local efficiency, 85 regions showed a linear increase with age (${\beta }_{{age}}\in [0.08,0.15]$, *p* < 0.05, Bonferroni corrected, Fig. 3b). For nodal degree centrality, 115 regions exhibited a significant age-related increase (${\beta }_{{age}}\in [0.07,0.19]$, *p* < 0.05, Bonferroni corrected, Fig. 3b). Of note, regions with a high rate of development were mainly located in the occipital cortex, fusiform gyrus, superior temporal gyrus, cingulate gyrus, hippocampus and precuneus. Furthermore, we also observed different developmental slopes of nodal properties, which followed posterior-to-anterior and inferior-to-superior gradients (Supplementary Fig. 2). Similar spatial patterns of development were observed when categorizing brain regions into 8 functional subnetworks according to Yeo’s brain parcellation^54^ (visual, somatomotor, dorsal attention, ventral attention, limbic, frontoparietal and default networks) and subcortical network within BNA246 template. Among the different networks, the visual network and somatomotor network had relatively higher ${\beta }_{{age}}$ than the other networks (Fig. 3c).

At the connectional level, the rich-club, feeder and local edges were categorized according to hub and nonhub regions (Supplementary Fig. 3a). The within-module or between-module edges were assigned based on a functional module architecture consisting of the 8 subnetworks (Supplementary Fig. 3b), and the long-range or short-range edges were classified by comparing the mean strength of the group-averaged network. When fitted with age, we calculated the connectivity strength changes of different types of edges after controlling for the global network strength. In Supplementary Fig. 3c-e, the connectivity strength of the local edge (${\beta }_{{age}}$ = 0.22, CI = [0.15,0.30], *t* = 5.64, *p* = 2.18E-08), within-module edge (${\beta }_{{age}}$ = 0.19, CI = [0.10,0.29], *t* = 4.12, *p* = 4.03E-05) and short edge (${\beta }_{{age}}$ = 0.22, CI = [0.15,0.29], *t* = 6.42, *p* = 2.17E-10) increased with age, while that of the between-module edge (${\beta }_{{age}}$ = −0.19, CI = [−0.31,−0.07], *t* = −3.09, *p* = 2.10E-03) and long edge (${\beta }_{{age}}$ = −0.38, CI = [−0.61,−0.15], *t* = −3.30, *p* = 1.00E-03) decreased with age. The connectivity strength of the feeder edge (${\beta }_{{age}}$ = −0.08, CI = [−0.20,0.04], *t* = −1.28, *p* = 0.20) and rich-club edge (${\beta }_{{age}}$ = −0.37, CI = [−0.77,0.02], *t* = −1.85, *p* = 0.07) remained stable with age. Additionally, the strength comparisons of different edges are shown in Supplementary Fig. 3f.

### Transcriptomic and cellular architectures of WM connectome development

**Fig. 4:**
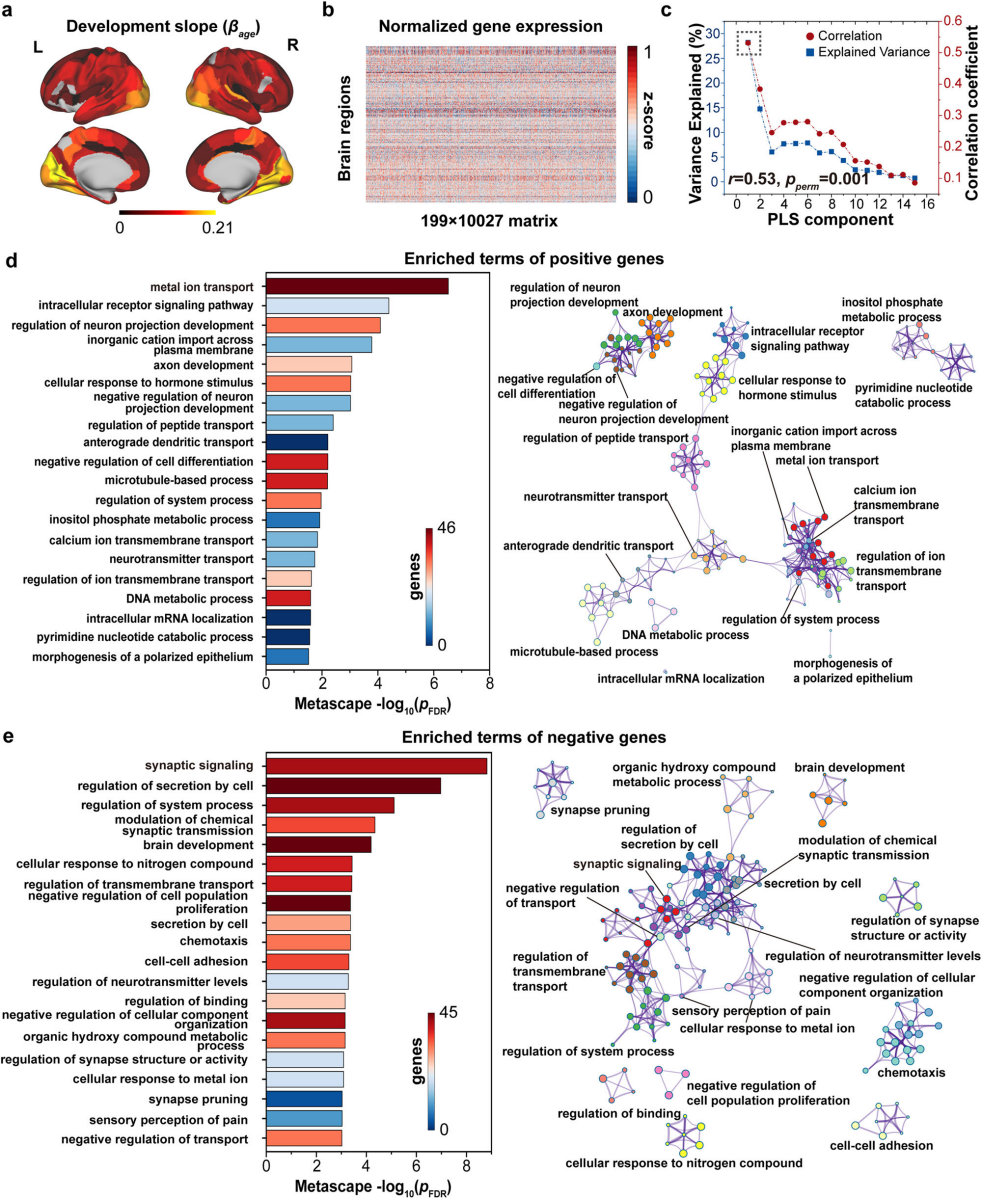
Association between the development slope of nodal efficiency and gene transcriptional profiles. **a** The map of standardized development slope (${\beta }_{{age}}$) in nodal efficiency across 199 brain regions. **b** The normalized gene transcriptional profiles comprised 10,027 genes in 199 brain regions, in which each row denotes the gene expression level for each gene in a brain region. **c** Explained ratios (left vertical axis) and correlation coefficients (right vertical axis) for the first 15 components obtained from PLS regression analysis. Enriched terms of positive genes (**d**) and negative genes (**e**). In **d** and **e**, the length of the bar denotes the enrichment significance and its color denotes the number of input genes falling under that term. Each circle node within network layout represented a term colored by cluster identity and its size denoted the number of genes falling into the term. The edge represents a similarity score between filtered terms.

To explore the potential transcriptomic association with WM connectome development, we employed AHBA^31^ along with a standardized processing pipeline^55^ to obtain spatial gene transcriptomic profiles across brain regions. Employing the partial least square (PLS) analysis^56^, we linked the spatial pattern (${\beta }_{{age}}$) of WM nodal efficiency development (Fig. 4a) and gene transcriptomic profiles (Fig. 4b). The gene expression score of the first PLS component accounted for the highest spatial variance explained at 28.2% (Fig. 4c). After spatial autocorrelation correction (SAC)^57^, there was a significant positive correlation (*r* = 0.53, *p* = 0.001, permutation test with SAC, Fig. 4c) between the first PLS component score of genes and the spatial pattern of WM regional development. Furthermore, we identified potential transcriptomic architectures in the GO biological process pathway^58^ using positive/negative genes with high weight on the first PLS component, respectively. The positive weight genes (771 genes) were prominently enriched for ion transport-related and development-related terms (*p* < 0.05, FDR corrected, Fig. 4d), such as “metal ion transport”, “regulation of peptide transport”, “regulation of neuron projection development”, and “axon development”. The negative weight (714 genes) gens were mainly enriched for synapse-related and brain development pathways (*p* < 0.05, FDR corrected, Fig. 4e), such as “synaptic signaling”, “synapse pruning”, and “brain development”.

**Fig. 5:**
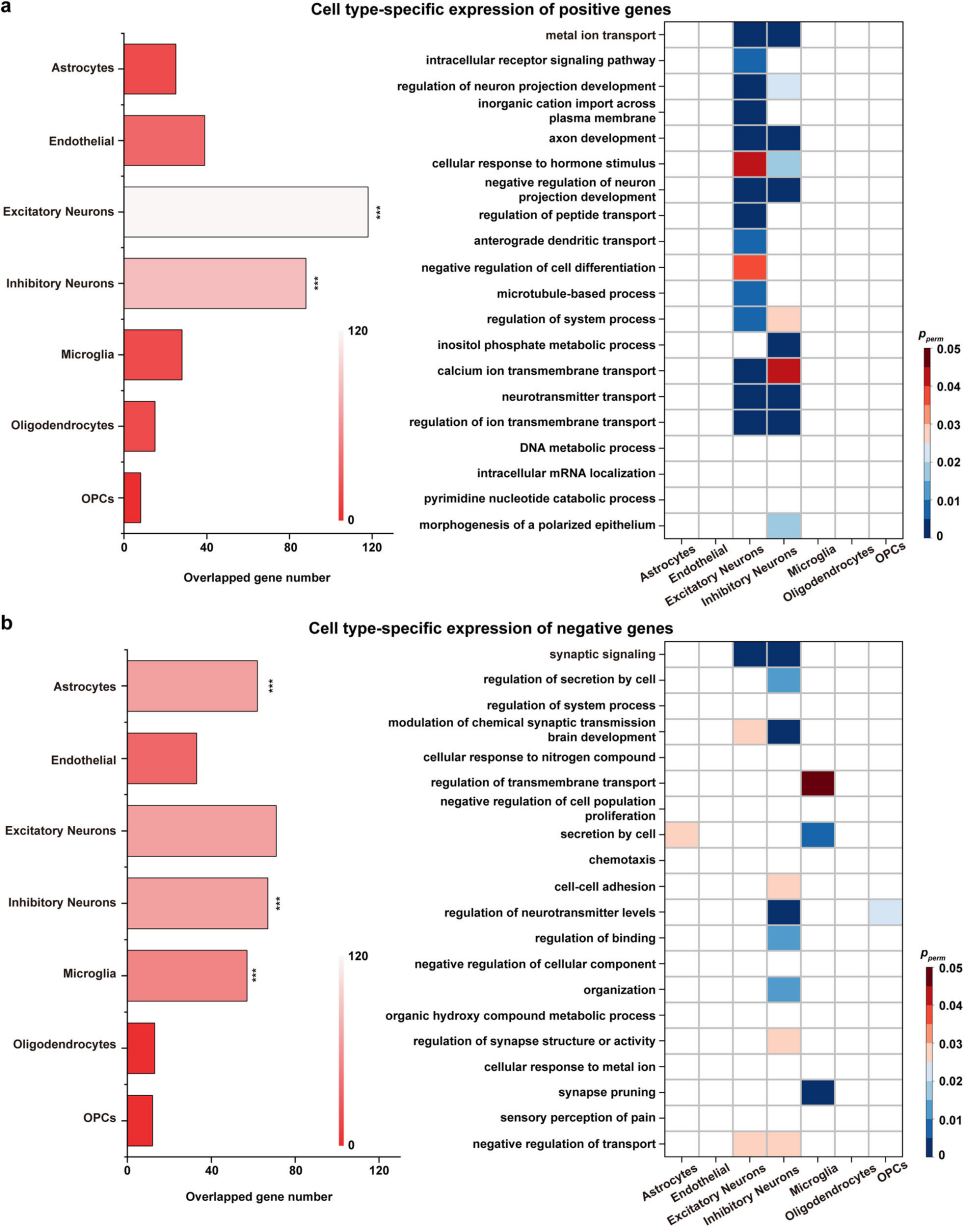
Cell type-specific analysis. Cell type-specific expression of positive genes (**a**) and negative genes (**b**). In **a** and **b**, the length and color of the bar shows overlapping numbers of the selected genes in each cell class. The color of the heatmap shows the statistically enriched terms in each cell class. Of note, OPCs: oligodendrocyte precursors. ***p < 0.001, permutation test.

To further investigate cell-specific expression of genes related to WM nodal efficiency development, the related genes were agglomerated into seven canonical cell classes^9,59–63^. These classes encompassed astrocytes, endothelial cells, excitatory neurons, inhibitory neurons, microglia, oligodendrocytes, and oligodendrocyte precursors. Our findings showed that the selected genes with high positive weights were significantly expressed in excitatory neurons and inhibitory neurons (118/88 genes, *p* < 0.001, permutation test, Fig. 5a). The genes with negative weights were expressed in astrocytes, inhibitory neurons and microglia (62/67/57 genes, *p* < 0.001, permutation test, Fig. 5b).

### Relationship to the cytoarchitecture of cortical organization

**Fig. 6:**
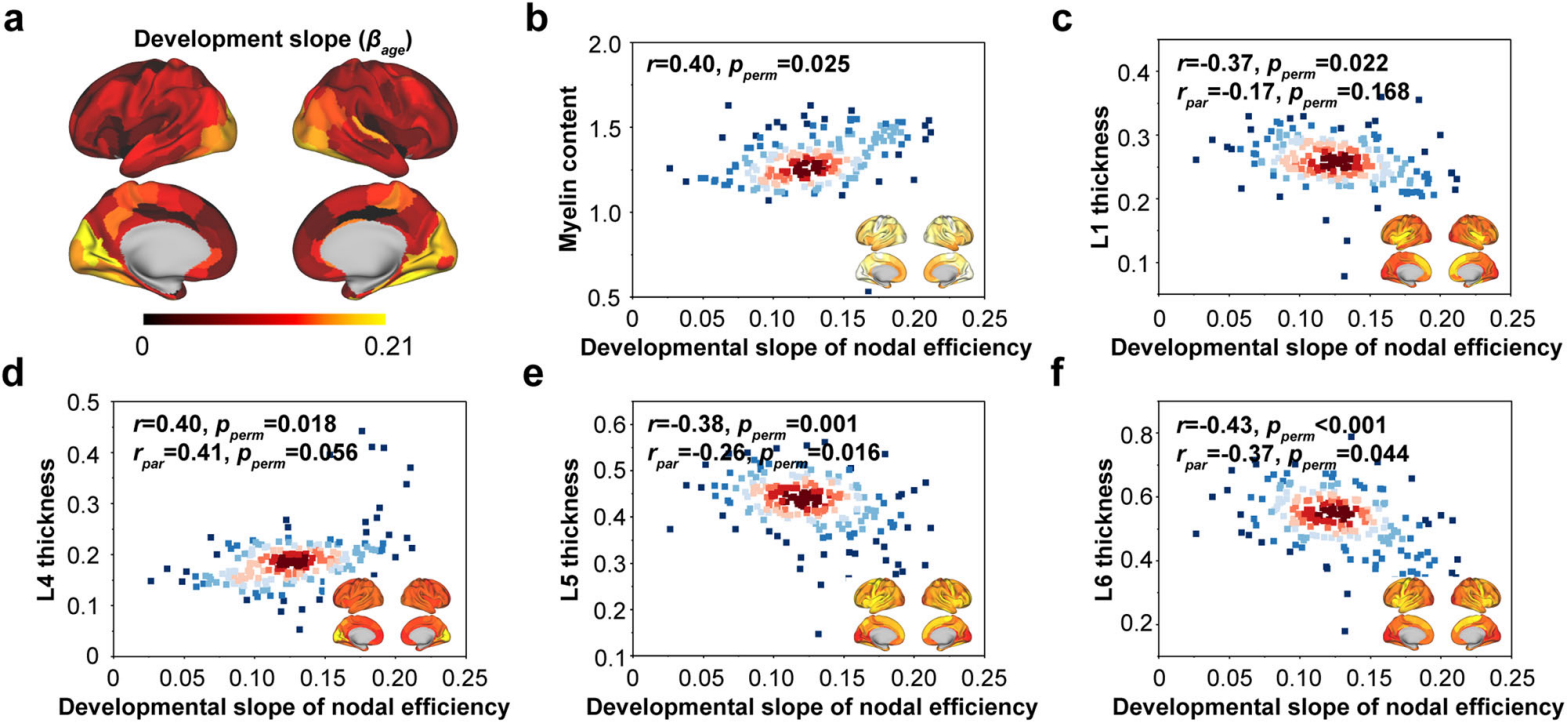
Developmental alterations of nodal efficiency align with cytoarchitecture of cortical organization. Developmental alterations in nodal efficiency (**a**) were significantly associated with myelin content (**b**) and laminar thickness of L1 and L4-L6 (**c**–**f**). The inset brain map in each panel is the pattern of fundamental properties of brain organization.

To assess the spatial correspondence between the developmental pattern of WM nodal efficiency and fundamental cytoarchitecture, we considered myelin content^43^ and the thicknesses of six cortical laminas (L1-L6) from the BigBrain atlas^41^. The high-resolution laminar thickness provided a more direct marker to map the relationship between WM nodal efficiency development and cytoarchitecture. We found that the spatial development pattern of WM nodal efficiency (Fig. 6a) was significantly associated with myelin content (*r* = 0.40, *p* = 0.025, permutation test with SAC, Fig. 6b) and L4 thickness (*r* = 0.40, *p* = 0.018, permutation test with SAC, Fig. 6d). In contrast, the nodes with higher development slopes tended to have lower thicknesses in the three laminas (L1: *r* = −0.37, *p* = 0.022, permutation test with SAC, Fig. 6c; L5: *r* = −0.38, *p* = 0.001, permutation test with SAC, Fig. 6e; L6: *r* = −0.43, *p* < 0.001, permutation test with SAC, Fig. 6f). After controlling effects of remaining laminas, L5 and L6 thicknesses showed specific positive correlations with the spatial development pattern of nodal efficiency, while L4 thickness exhibited a tendency of specific negative correlation (L4: *r*_*par*_ = 0.41, *p* = 0.056, permutation test with SAC, Fig. 6c; L5: *r*_*par*_ = −0.26, *p* = 0.016, permutation test with SAC, Fig. 6e; L6: *r*_*par*_ = −0.37, *p* = 0.044, permutation test with SAC, Fig. 6f). By directly calculating the cortical thickness on individuals, it was found that the development slope of brain region was negatively correlated with the group-average cortical thickness (*r* = 0.39, *p* = 0.036, permutation test with SAC, Supplementary Fig. 4).

### Reproducibility analyses

We assessed the consistency of the results by incorporating head movement as an additional covariate. Briefly, we integrated head movements as an additional covariate within the mixed effect model to assess the developmental changes of global and nodal properties. The normalized gene weights were derived from PLS correlation between ${\beta }_{{age}}$ of nodal efficiency and gene expression. At the global level, global efficiency, local efficiency, and network strength exhibited a significant positive correlation with age, and the shortest path showed a significant negative correlation with age (*p* < 0.05, Bonferroni corrected, Supplementary Fig. 5a). At the regional level, the developmental slope ${\beta }_{{age}}$ across nodal efficiency, local efficiency and degree centrality were consistently significant, aligning with the results from the original model (Supplementary Fig. 5b, c). The normalized gene weights demonstrated consistency between the two models, based on the significance of the correlation (*r* = 0.998, *p* < 0.001, permutation test with SAC, Supplementary Fig. 5d).

We examined whether changes in the WM connectome were robust to distinct brain parcellation templates. Using the Automated Anatomical Labeling with 90 brain regions (AAL90)^64^, we repeated the network construction and analysis procedures. At the global level, several global network metrics, including global efficiency, local efficiency, and network strength exhibited a significant positive correlation with age, and the shortest path showed a significant negative correlation with age (*p* < 0.05, Bonferroni corrected, Supplementary Fig. 6a). At the regional level, the spatial distributions of the regions with significant age-related alterations were similar to the results from BNA246 template, which were mainly distributed across the occipital cortex, fusiform gyrus, superior temporal gyrus, cingulate gyrus, hippocampus and precuneus (Supplementary Fig. 6b). To examine sex differences, similar results were found between the male and female groups and none of properties with significant sex differences were observed (*p* > 0.05, Bonferroni corrected).

We proceeded to create networks weighted by FN, FA, and the inverse of mean diffusivity (1/MD) employing the BNA246 template. These networks were formulated to systematically assess the resilience of diverse connection-weighting approaches in delineating WM connectomes, complementing the principal findings. At the global level, various weighting strategies exhibited similar outcomes (Supplementary Table 3-5). At the regional level, spatial distributions were similar to those observed in the FN×FA-weighted network outcomes (Supplementary Fig. 7a–c), with the FN-weighted network demonstrating greater consistency than that of FA and 1/MD (Supplementary Table 7).

We constructed an additional FN×FA-weighted network using deterministic tractography based on a ball-and-stick model^65^. The validation of global and nodal properties is detailed in Supplementary Table 6 and illustrated in Supplementary Fig. 7d. Additionally, a significant correlation was established with the nodal outcomes of a tensor model-based FN×FA-weighted network (Supplementary Table 7). Through PLS analysis, we identified a significant positive correlation (*r* = 0.46, *p* < 0.001 permutation test with SAC, Supplementary Fig. 8a) between the first PLS component score of genes and the ${\beta }_{{age}}$ of nodal efficiency. The gene analysis results remained consistent, contingent on the significance of the correlation (*r* = 0.94, *p* < 0.001, permutation test, Supplementary Fig. 8b) of the normalized gene weight. Notably, the positively correlated genes were predominantly enriched for transport- and development-related terms, while the negatively correlated genes exhibited significant enrichment for synapse- and development-related terms (Supplementary Fig. 8c,d). In cell-specific analysis, genes positively correlated expressed in excitatory neurons and inhibitory neurons, whereas negatively correlated genes expressed in astrocytes and microglia (*p* < 0.01, permutation test, Supplementary Fig. 8e, f). We additionally found that the gene association and cell specificity results based on probabilistic tractography exhibited agreement with the original results as shown in Supplementary Fig. 9.

Utilizing the ABAnnotate toolbox^66,67^, which considered both gene coexpression and spatial autocorrelation, we validated the enrichment results regarding the spatial correlation between the developmental slope ${\beta }_{{age}}$ of WM nodal efficiency and gene expression profiles. The results revealed that the majority of the initially identified functional pathways remained significantly enriched, as illustrated in Supplementary Fig. 10.

Finally, we also evaluated whether our connectomic and transcriptomic findings could be replicated in an independent development cohort (HCP-D). The changes of global properties were consistent with those of the CBD cohort (Supplementary Table 8). For developmental alterations in nodal efficiency (Supplementary Fig. 11a), a significant correlation (*r* = 0.43, *p* < 0.001, permutation test with SAC, Supplementary Fig. 11b) existed across nodal age-related changes between two distinct cohorts, indicating that the heterogeneous spatial development found in this study was robust during childhood and adolescence. The findings of the gene analysis were consistent between two cohorts, depending on the significance of the correlation (*r* = 0.80, *p* < 0.001, permutation test, Supplementary Fig. 11c) of the normalized gene weight. The selected positive genes were mainly enriched for transport-related terms, while the negative genes were significantly enriched for development-related terms, as shown in Supplementary Fig. 11d. In cell-specific analysis, the positively correlated genes expressed in excitatory neurons, and the negatively correlated genes expressed in astrocytes (*p* < 0.001, permutation test, Supplementary Fig. 11e). We also replicated a tendency that the spatial development pattern of global efficiency is associated with L4 thickness (*r* = 0.32, *p* = 0.04, permutation test with SAC) but not with other fundamental properties.

## Discussion

This study performed a multiscale evaluation of WM connectome development from childhood to adolescence. Using a large longitudinal cohort aged 6 to 13 years old of up to 3 times of MRI scans, we observed a linear increase in brain network efficiency with increasing age, and more rapid development were found mainly in the occipital cortex, fusiform gyrus, superior temporal gyrus, cingulate gyrus, hippocampus and precuneus. Moreover, we found that the spatially heterogeneous development of the WM connectome was associated with transcriptomic architectures. Specifically, the positive genes were enriched in transport-related and development-related pathways, with significant expression in excitatory neurons and inhibitory neurons. The negative genes were enriched in synapse-related and development-related pathways, relating to astrocytes, microglia, and inhibitory neurons. Additionally, we demonstrated that the heterogeneous development was related to the myelin content and laminar thickness properties of cortical organization, providing microscopic evidence for the underlying mechanisms at the gene and cell levels. Together, our study characterized the age-related trajectory of WM connectome development from childhood to adolescence and investigated whether its heterogeneous development is associated with transcriptomic architecture, cellular organization, or cortical properties.

From childhood to adolescence, the WM connectome exhibits enhanced integration capacity that can be characterized by increases in global efficiency, local efficiency, and network strength with age. These findings not only are consistent with previous findings mainly from cross-sectional studies and small-sample longitudinal studies^5,17–20^ but also extend to longitudinal evidence of higher statistical power with the large-sample, and multiple assessments^68^. Our results also revealed a heterogeneous development layout in nodal network properties, with primary regions (e.g., visual cortex, sensorimotor cortex) showing more rapid growth in nodal efficiency than other regions. From the perspective of structure-function coupling, the development of WM structural connectivity promoted the maturation of functional specialization^47–50^. Combing with findings from previous studies of early childhood^69^ and late adolescence^70^, this study particularly suggested that the rapid development in the WM connectome of the primary cortex between 6 and 13 years may support the subsequent development of higher-order cognitive functions. Furthermore, we observed a broad pattern of heterogeneous development across brain regions along posterior-to-anterior and inferior-to-superior gradients, in line with findings of previous WM microstructure studies of development^71,72^. More changes in medial than lateral regions for local efficiency and degree centrality were also in agreement with an FA study of the WM skeleton^71^. Therefore, the development of the WM connectome may also follow major gradients in the brain. Neurodevelopmental changes in network connections are characterized by simultaneous progressive and regressive changes^13^. Likewise, our results demonstrated a developmental pattern of WM connections characterized by integration and segregation. Additionally, we observed a strengthening of connectivity in local, within-module and short-distance edges, as well as a pruning of connectivity in between-module and long-distance edges. Such findings suggest that over the course of development, the WM connectome is dominated by spatial increases in intramodule connections, along with the refinement of intermodule connections. The connectivity of the feeder edge and rich-club edge remained stable with age, highlighting the stability of the hubs structure that supports the enhancement of complex information integration during this period^16,73,74^. In recent years, the topology of WM functional connectome have been gradually studied^75–77^. Comparative studies examining both functional and structural integration and separation of WM networks, alongside the consideration of mental illness, could provide valuable insights into the neurodevelopmental processes and disease mechanisms associated with the WM network.

The AHBA^31^ has been pivotal in bridging the gap between neuroimaging and transcriptomics^32^. The analyses of regional expression has proven valuable in identifying associations between regional gene variations and some regional properties^32,78^. Earlier studies in mouse and rat brains have uncovered correlations between regional gene profiles and nodal degree centrality as well as participation coefficients^79,80^. In human brain studies, spatial correlations between transcriptome patterns and WM network disconnection patterns have been leveraged to identify pathologically associated genes^38,81^. A recent study utilized spatial patterns of nodal degree centrality for correlating transcriptome patterns as a methodological validation^67^. These collective findings give rise to a common hypothesis suggesting that genes in the cortex of corresponding locations influence the nodal properties of the WM connectome. Additionally, the WM network efficiency is considered a key aspect of brain maturation, crucial for information processing and cognitive functions^20^. The heritability of global efficiency during adolescence has been demonstrated by twin studies^18,70^. In light of these contexts, we regarded the pattern of developmental changes in nodal efficiency of cortical regions as a brain phenotype, seeking to identify gene associations within the transcriptome of cortical regions.

Our results showed that positively correlated genes were enriched for ion transport-related and development-related pathways, while negatively correlated genes were enriched for synapse-related and development-related pathways. Interestingly, the finding of ion transport we identified coincided with previous studies of cortical structural connectome development^35^ and functional connectome development^36^. Ion transport balances intracellular and extracellular concentration difference to stabilize brain neural circuits^82,83^. It is speculated that ion transport-related gene pathways may regulate development in the brain connectome by maintaining and enhancing network stability. Synaptogenesis (especially synaptic pruning) is considered critical for brain connectome specificity during childhood and adolescence^84,85^. Notably, genes related to ion channels and synapses have been found to shape neuronal timescales, which are associated with higher-order cognitive functions, such as memory, decision making, and reasoning^86^. Axon development and neuron projection development^87^ were important pathways directly associated with enhanced and refined changes in WM connectome through processes such as axonal fasciculation and defasciculation. Our findings suggested that the transport-related, synapse-related, and development-related pathways may regulate gradual integration and differentiation in WM connectome from childhood to adolescence, thus laying the foundation for their cognitive and learning development.

Additionally, we investigated cell-specific types in spatial gene expression of WM connectome development. We found that the positively correlated genes related to WM connectome development were significantly expressed in excitatory neurons and inhibitory neurons also known as glutamatergic neurons and GABAergic neurons, respectively. These neurons have different neuronal subsets and projection patterns, which jointly constitute a homeostatic regulatory mechanism of the brain connectome to control signal flow, sculpt network dynamics^88,89^, and regulate different behavioral functions^90^. A recent study of developmental neuroplasticity markers found that the decreased cortical excitation-inhibition ratio is driven by the pruning of excitatory neurons and the maturation of GABAergic neurons^91^. In contrast, the negatively correlated genes were expressed in astrocytes, inhibitory neurons, and microglia. Both astrocytes and microglia have been implicated in synaptic pruning, or the elimination of weak and inappropriate synapses, a critical developmental process for the formation of fully functional neuronal circuits^92–94^. Notably, the specific and precise expression of synapses is conducive to the establishment of intercellular connection patterns of GABAergic neurons^95^. From childhood to adolescence, learning and the environment factors can drive changes in the brain while maintaining a balance of brain activity is key to constant fine-tuning of the brain^96,97^. Learning-related adaptations are encoded as changes in synaptic strength or other cellular properties^97^ and may be further refined through synaptic pruning^94^, ultimately resulting in the precise wiring of mature neural circuits. This process is particularly important for cortical plasticity in children and adolescents^98^. Therefore, genes associated with the developmental WM connectome exhibit specific expression patterns in cellular organization, may be closely related to the construction and maintenance of connectomic homeostasis within the brain during learning and development. Nevertheless, the associated physiological mechanisms will require further study.

One crucial question concerns whether the heterogeneous development of the WM connectome across regions can reflect fundamental properties of cortical organization. We found that the spatial development of the WM connectome conformed to the myelin content, which is present in most long-distance projection neurons and supports the enhancement of the neural signal-to-noise ratio and the coordination of distributed neural activity^43^. Histologically, cortical regions that are more heavily myelinated generally tend to be thinner^43^. The spatial pattern of accelerated cortical thinning from childhood to adulthood is associated with increased expression of genetic markers related to inhibitory and excitatory neurons, with enriched axon-related terms (e.g., axonal development)^99^, further supporting our findings at the gene and cell levels. We also found that the spatial development of the WM connectome was inversely associated with the cortical thickness of L1, L5, and L6, with L5 and L6 contributing to overall thickness gradients and sending information externally^100^. In contrast, L4 may be involved in the reception, integration, synchronization, and regulation of sensory peripheral signals in the human cortex and extracortex^84,101^. Interestingly, the cortical thickness of L4 had a positive association with the development of the WM connectome, indicating that the transfer function of L4 is enhanced in conjunction with the transmission efficiency of the developing WM connectome. A recent study has proposed that glutamatergic pathways between the cortex and thalamus transmit information to L4 through transthalamic circuits, and from L4 to other laminas via internal intercellular communication^102^. In the rat barrel cortex, L4 has averaged 62% more GABA contacts per unit volume than any other cortical layer^103^, and the axonal projection of spiny L4 neurons highly associates with the structure of a cortical column^104^. These findings highlight that the cortical differentiation microstructure underpins the developmental of the WM connectome and predict that the association may be dominated by genes that tend to regulate neuronal cell proliferation, differentiation, and migration.

Several methodological issues should be addressed. First, the observed developmental trajectories in the WM connectomes could be influenced by the age range of 6 to 13 years in this study. Conducting future longitudinal studies across a wider age spectrum will enhance the precision of WM connectome trajectories. Second, although we contributed by uncovering the transcriptomic architecture of the spatial development pattern of the WM connectome using the AHBA, it is essential to acknowledge that the results might miss the main factor of development due to indirect gene association and variable gene expression during development. Nevertheless, a study found that the relative spatial patterns of genes did not change much after birth^8^. Third, the network topologies usually involve different structures, including cortical regions and extra-cortical WM pathways. This raises the possibility that results associated with gene expression in cortical regions may reflect the overall gene effects on different structures. Moreover, the construction of WM network is constrained by issues like directionality and time variability, which could impact spatial gene association results. Addressing these challenges will necessitate more reasonable assumptions and advanced methods to broaden our understanding of the spatial transcriptome patterns of WM connectome. Fourth, a multivariate paradigm that encompasses gene–brain–behavior–environment has been advocated to understand the complex neurodevelopmental processes of growth and specialization that modify the brain to adapt to the environment^105^. Future research should incorporate this paradigm to further refine our findings in WM connectome development. Fifth, coupling studies of WM and functional connectomes revealed that brain function is structurally constrained by WM structure^47,48,50^. Therefore, future longitudinal studies combining multimodal connectomes will provide a more comprehensive view of the developmental process during this period. Finally, validating the sensitivity of the analytical pipeline and ensuring compatibility with independent longitudinal data is crucial. In our study, we took steps to validate our main findings by employing different network construction^106,107^ and gene association^67^ methods. We observed that the choice of pipeline had some impact on the subsequent findings, underscoring the importance of methodological guidance in studies of this nature. Additionally, we obtained a moderate correlation of a main finding using a cross-sectional cohort from the HCP-D, which can arise from a variety of factors including ethnicity, environment, the paradigm of data acquisition, etc., which also emphasizes the importance of research harmonization.

In conclusion, we have demonstrated the multiscale development pattern of the WM connectome from childhood to adolescence. The spatially heterogeneous development of WM connectivity was regulated by transcriptomic architectures. In particular, positively correlated genes contribute to the cellular organization of excitatory and inhibitory neurons, while negatively correlated genes relate to astrocytes, inhibitory neurons, and microglia. Additionally, the heterogeneous development of the WM connectome was associated with myelin content and the thicknesses of specific lamina of the cortex. Therefore, our findings may offer insights into understanding the normal development of the brain connectome and plasticity, which may provide clues for the early diagnosis and treatment of development-related brain disorders.

## Methods

### Participants

We used a cohort from the CBD^44^, an ongoing longitudinal dMRI study, in the present study. From the cohort, typically developing children were recruited from Beijing primary schools. The exclusion criteria included the presence of intellectual or developmental abnormalities, a history of neurological or psychiatric disorders, the use of psychoactive drugs, and the presence of a significant head injury. All the participants underwent at least one MRI acquisition at three-time points 1 year apart. A total of 604 typically-developing children (age range of 6 to 13 years, 339 males and 266 females), including 1033 scans were selected for analysis in the present study (Supplementary Table 1) after age matching and quality control during MRI preprocessing. This study was conducted according to the guidelines of the Declaration of Helsinki and was approved by Beijing Normal University Institutional Review Board. Informed consent was obtained from parents/guardians of all participants.

### Imaging acquisition and preprocessing

The MRI data were acquired using the same Siemens Trio 3 T scanner with a 16-channel phased array head coil at the Beijing University center and the Beijing Huilongguan Hospital center. MRI scanning included the collection of 3D T1-weighted structural MRI with a 1 mm^3^ isotropic voxel size (TR = 2530 ms, TE = 2.98 ms, TI = 1100 ms, flip angle = 7°, FOV = 256 × 224 mm^2^, and 192 sagittal slices) and diffusion-weighted MRI (DWI) with a 2 mm^3^ isotropic voxel size (64 diffusion directions with b = 1000 s/mm^2^ and 10 images with *b* = 0 s/mm^2^, TR = 7500 ms, TE = 64 ms, flip angle = 90°, FOV = 224×224 mm^2^, and 70 axial slices). The preprocessing procedures for dMRI data comprised the correction of the eddy current and motion artefacts, the estimation of the diffusion tensor elements, and the calculation of the fractional anisotropy (FA). The eddy current distortions and motion artefacts in the dMRI data were corrected by applying an affine alignment of each DWI image to the b0 image. Then the diffusion tensor elements were estimated by solving the Stejskal and Tanner equations, and the FA value of each voxel was calculated. All procedures were executed using the FMRIB’s Diffusion Toolbox of the FMRIB Software Library (https://fsl.fmrib.ox.ac.uk/fsl/fslwiki/FDT).

### Image quality control

Rigorous quality control was conducted for T1 and dMRI images. An experienced radiologist examined each T1 image to ensure the absence of arachnoid cysts, neuroepithelial cysts, or any other intracranial occupying lesions. Subsequently, five trained raters visual inspected the T1 images for brain damage, missing layers, or evident noise. Out of the original 1072 T1 images, 32 were excluded due to poor image quality, leaving 1040 images entering the subsequent analysis. For dMRI image, images reported as failures by DTIprep^108^ were excluded. Additionally, visual inspections by five trained raters were conducted, and images with abnormal volume proportions exceeding 10% were excluded. Out of the 1053 dMRI images initially acquired, 1033 images passed the quality control. Finally, 1033 scans with both T1 and dMRI images were included in the subsequent analysis.

### WM network construction

The BNA246^51^ template was used to define network nodes. Briefly, a b0 image was first aligned to a native T1 image, and then the native T1 image was normalized to an asymmetric T1 template for 6-12 years from Chinese Paediatric Atlases^109^ using the FMRIB Software Library (https://fsl.fmrib.ox.ac.uk/fsl). Inverse transformation matrices derived from the aforementioned steps were applied to transform the brain atlas of standard space into native space. Following our previous methodological evaluation study^107^, the dMRI data with single b value is suitable for a deterministic tractography with a single tensor model to reconstruct whole-brain fiber tracts based on the Diffusion Toolkit (http://www.trackvis.org/dtk/). Based on the tractography results, the FA$\times$FN-weighted network of each participant was constructed, where the FA$\times$FN weight was defined as the average FA value of the voxels traversed along the connected fibers between two regions times the number of fiber streamlines (FN) connecting two brain regions. Two regions are deemed structurally connected if there is at least one streamline fiber present, with both of its end-points located within these two regions^24^.

### Global network properties

Leveraging graph theory model, network properties can be derived to reflect the brain’s various characteristics. Eight whole-brain properties were calculated according to the constructed network, including global efficiency, local efficiency, shortest path, network strength, clustering coefficient, and small-world parameters ($\gamma$, $\lambda$ and $\sigma$)^13^.

The global efficiency measures the efficiency of parallel information transfer in the whole network $G$^110^, which can be computed as:

1 $${E}_{{glob}}\left(G\right)=\frac{1}{N}{\sum }_{i\in G}\frac{{\sum }_{j\in G,j\ne i}{d}_{{ij}}^{-1}}{N-1}$$

where ${d}_{{ij}}$ is the shortest path length between node $i$ and node $j$ in $G$. $N$ is the number of nodes in $G$.

The local efficiency of $G$ reveals how much the network is fault tolerant, showing how efficient the communication is among the first neighbors of the node $i$ when it is removed. The local efficiency of a graph is measured as:

2 $${E}_{{loc}}(G)=\frac{{\sum }_{i\in G}{E}_{{glob}}({G}_{i})}{N}$$

where ${G}_{i}$ denotes the subgraph composed of the nearest neighbors of node $i$.

The shortest path of a network quantifies the ability for information to propagate in parallel. The shortest path length of a network was computed as follows:

3 $${L}_{p}\left(G\right)=\frac{{\sum }_{j\in G,j\ne i}{d}_{{ij}}}{N\left(N-1\right)}$$

where the shortest path length ${d}_{{ij}}$ between any pair of nodes (e.g., node $i$ and node $j$) is defined as the sum of the edge lengths along this shortest path. For weighted networks, the length of each edge was assigned by computing the reciprocal of the edge weight ($1/{w}_{{ij}}$).

The network strength quantifies the overall connectivity within the brain network. For a network $G$, the strength of $G$ was calculated as:

4 $${S}_{p}\left(G\right)=\frac{{\sum }_{i\in G}S\left(i\right)}{N}$$

where $S(i)$ is the sum of the edge weights ${w}_{{ij}}$ linking to node $j$. And the strength of a network is the average of the strengths across all of the nodes in this network.

The clustering coefficient indicates the extent of the local interconnectivity or cliquishness in a network^111^, and calculated as:

5 $${C}_{p}\left(G\right)=\frac{{\sum }_{i\in G}{C}_{p}\left(i\right)}{N}$$

where $C(i)$ is the likelihood of whether the neighborhoods of node $i$ were connected with each other or not, and is computed as follows:^112^

6 $${C}_{p}\left(i\right)=\frac{2\times {\sum }_{j,l\in G}{\left({\bar{w}}_{{ij}}{\bar{w}}_{{jl}}{\bar{w}}_{{li}}\right)}^{\frac{1}{3}}}{{k}_{i}\left({k}_{i}-1\right)}$$

where ${k}_{i}$ is the degree of node $i$ and $\bar{w}$ is the weight of edge, which is scaled by the largest weight of the network. Of note, the clustering coefficient is zero if the nodes are isolated or have just one connection.

The small-world network exhibits a high level of clustering close to regular networks, while still maintaining a short average path length close to random networks. The clustering coefficient and the shortest path length of the brain networks were compared with those of random networks. In this study, we generated 5,000 matched random networks, which had the same number of nodes, edges, and degree distribution as the real networks^113^. Furthermore, we computed the normalized $\gamma$ and $\lambda$ as follows:

7 $$\gamma =\frac{{C}_{p}^{{real}}}{{C}_{p}^{{rand}}}$$

8 $$\lambda =\frac{{L}_{p}^{{real}}}{{L}_{p}^{{rand}}}$$

where ${C}_{p}^{{rand}}$ and ${L}_{p}^{{rand}}$ are the mean ${C}_{p}$ and the mean ${L}_{p}$ of 5,000 matched random networks, respectively. A real network would be considered small-world if $\gamma > 1$ and $\lambda \approx 1$^111^. Thus, the small-worldness $\sigma$ can be defined as follows:

9 $$\sigma =\frac{\gamma }{\lambda }$$

where $\sigma$ is typically greater than 1 for small-world networks^114^.

### Local network properties

For each brain region, four common nodal properties were calculated: nodal efficiency, nodal local efficiency, nodal degree centrality, and nodal betweenness centrality^13^.

The nodal efficiency^115^ quantifies the nodal contribution to the overall efficiency of communication across the entire network, which can be calculated using the following equation:

10 $${{nE}}_{{glob}}\left(i\right)=\frac{{\sum }_{j\in G,j\ne i}{d}_{{ij}}^{-1}}{N-1}$$

where n is the number of nodes and ${d}_{{ij}}$ is the shortest path length between nodes $i$ and $j$.

The nodal local efficiency^115^ quantifies the nodal contribution to the local communication efficiency, which can be calculated as:

11 $${{nE}}_{{loc}}\left(i\right)={E}_{{glob}}\left({G}_{i}\right)$$

The nodal degree centrality quantifies the total number/strength of the connections of one node in the network:

12 $${k}_{i}=\mathop{\sum} \limits_{j\in N}{w}_{{ij}}$$

The nodal betweenness centrality^116^ quantify the role of a node in facilitating communication between other node pairs in the network. The nodal betweenness centrality of node $i$ was defined as:

13 $${b}_{i}=\frac{1}{(n-1)(n-2)} \mathop{\sum} \limits_{{j,k\in G}\atop{j\ne k,k\ne i,i\ne j}}\frac{{\rho }_{{jk}}(i)}{{\rho }_{{jk}}}$$

where ${\rho }_{{jk}}$ is the number of shortest paths between node $j$ and node $k$, and ${\rho }_{{jk}}(i)$ is the number of shortest paths between node $j$ and node $k$ passing through node $i$.

### Functional subnetwork properties

From the perspective of the functional subnetwork, brain regions were assigned to seven different functional networks^54^ according to an official corresponding table provided by the official website (http://www.brainnetome.org/), and subcortical regions within BNA246^51^ were defined as the subcortical function network. Furthermore, the properties of different functional networks were the average properties of assigned regions.

### Connection properties

Various measures of centrality enable the identification of central brain hubs characterized by high-degree connectivity. To reduce false-positive edges, one edge was zero if its nonzero number was less than 75% at the group level^117^. Then, one node of the group-averaged network was defined as the hub if its nodal degree centrality or nodal betweenness centrality was greater than the *mean* $\pm \,$*std* of all nodes; otherwise, it was defined as a nonhub. According to the different categories of two nodes, the existing edges between them were classified into three types: local (nonhub to nonhub), feeder (hub to nonhub) and rich-club (hub to hub)^16^.

The brain networks have a pronounced tendency to form functional modules, reflected by an abundance of connectivity within each module and the relatively sparse connectivity between modules. Based on the functional modular architecture consisting of the 8 subnetworks, the edges of all participations were assigned as two types: within-modular edge and between-modular edge.

The physical distance of streamline fibers was defined as the average length of all streamline fibers between two regions, removing the effects of brain size. Edge length in the group-averaged network was the average of the lengths of corresponding edges across individual participants. The threshold was calculated as the average of all edge lengths to define two edge types: short edge and long edge.

### Age-related trajectory of WM network organization

To characterize developmental trajectories of various properties of WM network organization, a mixed effect model^52^ was applied to mine the intrinsic longitudinal relationship between properties and age in our study. For each measure, both linear and quadratic models were constructed after controlling for total brain volume, center, and sex.

The linear model was as follows:

14 $${y}_{{ij}}= 	 \,{\beta }_{0}+{b}_{i}+\left({\beta }_{{age}}+{b}_{{age},i}\right){{age}}_{{ij}}+{\beta }_{{sex}}{{sex}}_{i} \\ 	 +{\beta }_{{tbv}}{{tbv}}_{{ij}}+{\beta }_{{centre}}{{centre}}_{i}+{\varepsilon }_{{ij}}$$

where ${y}_{{ij}}$ is the network measures of participation $i$ at the $j$ time point, ${\beta }_{{age}}$ is the fixed effect, ${b}_{{age},i}$ is the random effect of participation $i$, ${{age}}_{{ij}}$ is the acquisition age of participation $i$ at the $j$ time point. Total brain volume (${{tbv}}_{{ij}}$), center (${{centre}}_{i}$) and sex (${{sex}}_{i}$) are considered as covariates. ${\varepsilon }_{{ij}}$ is the residual of participation $i$ at the $j$ time point.

The quadratic model was as follows:

15 $${y}_{{ij}}= 	 \,{\beta }_{0}+{b}_{i}+\left({\beta }_{{{age}}^{2}}+{b}_{{{age}}^{2},i}\right){{{age}}_{{ij}}}^{2}+\left({\beta }_{{age}}+{b}_{{age},i}\right){{age}}_{{ij}}\\ 	 +{\beta }_{{sex}}{{sex}}_{i}+{\beta }_{{tbv}}{{tbv}}_{{ij}}+{\beta }_{{centre}}{{centre}}_{i}+{\varepsilon }_{{ij}}$$

where ${\beta }_{{{age}}^{2}}$ is the fixed effect and ${b}_{{{age}}^{2},i}$ is the random effect of participation $i$.

For sex difference, a linear model which included age-by-sex interaction term was utilized as follows:

16 $${y}_{{ij}}= 	 \,{\beta }_{0}+{b}_{i}+\left({\beta }_{{age}}+{b}_{{age},i}\right){{age}}_{{ij}}+{\beta }_{{sex}}{{sex}}_{i}\\ 	 +{\beta }_{{age}* {sex}}\left({{age}}_{{ij}}* {{sex}}_{i}\right) +{\beta }_{{tbv}}{{tbv}}_{{ij}}+{\beta }_{{centre}}{{centre}}_{i}+{\varepsilon }_{{ij}}$$

Notably, the network strength was included as a covariate in edge analysis to delineate intrinsic edge topology alteration^21,47^. The Akaike information criterion^53^ was used to determine the optimal model, with a lower value reflecting a trade-off between the likelihood and simplicity of a model. The Markov Chain Monte Carlo method estimated the standard error and 95% confidence interval (CI) of age effect. In local and functional subnetwork level models, nodal and functional subnetwork measures were z-score standardized to obtain a standard slope for facilitating comparisons of different measures. The *p* values of models were corrected for multiple comparisons by the Bonferroni method.

### Main gradients of the developmental slope

To explore how developmental slope varied along various gradients, we extracted the mean slope of each nodal property across all regional voxels for posterior-to-anterior, medial-to-lateral or inferior-to-superior slice including more than 500 voxels. Then we tested whether a change was significantly different along gradients by comparing slopes between two group slices separated by a midline of the brain. Of note, medial-to-lateral slices were separated into the lateral group and medial group in every hemisphere. The *t*-test was used to verify its significance with *p* < 0.05.

### Association between WM development and gene expression

For the AHBA dataset, the preprocessing of anatomic and genomic information was performed by referencing a recommended pipeline^55^. Specifically, we generated preprocessed structural data by FreeSurfer (https://surfer.nmr.mgh.harvard.edu/fswiki/) for each donors. According to official scripts (http://www.brainnetome.org/resource/), the BNA246 template was projected on native fsaverage space. Finally, an averaged gene expression profile of 10,027 genes covering 199 cortical regions (excluding 47 cortical regions that had an insufficient number of assigned samples) was produced.

PLS correlation^56^ was performed to mine the weighted linear combinations (components) of gene expression profiles associated with the spatial development slopes ${\beta }_{{age}}$ of the WM connectome. Specifically, we utilized development slopes ${\beta }_{{age}}$ from 199 brain regions that spatially matched with the gene expression profile. For each PLS component, We calculated Pearson’s correlation coefficient to assess the association between the PLS score and development slopes ${\beta }_{{age}}$. To correct for spatial autocorrelation, we compared the empirically observed value with spatially constrained null models generated by 10,000 permutations of surrogate maps of development slopes ${\beta }_{{age}}$^57^. Furthermore, we transformed the gene weight into a z-score value by dividing the standard deviation of the corresponding weights estimated from bootstrapping and ranked all genes. The significant genes with a Bonferroni of 1% were identified for the positive or negative gene list. Then, we performed gene functional enrichment for the GO biological process pathway search with Metascape^58^, focusing on selected high positive or negative genes. The resulting enrichment pathways were retained for significance at FDR < 0.05. Briefly, enriched terms were filtered by calculating accumulative hypergeometric *p* values and enrichment factors, and then hierarchically clustered into a tree according to Kappa similarity among their gene memberships. A threshold value kappa score of 0.3 was applied to cast the tree into term clusters.

### Cell type-specific analysis

The selected genes were initially assigned to 58 cell types derived from five studies focusing on single-cell research using the human postnatal cortex^9,59–62^, and these cell types were subsequently integrated into seven canonical classes^63^. Specifically, the cell classes comprised of astrocytes, endothelial cells, excitatory neurons, inhibitory neurons, microglia, oligodendrocytes, and oligodendrocyte precursors. The method avoided possible bias including acquisition methodology, analysis, or threshold method^63,118^. For statistics of cell types, we calculated overlapping numbers of the selected positive/negative genes in each cell class. A null model was generated by 10,000 random resamples in genes within each cell type to test the significance of the results. In addition, the genes involved in each enriched term were subjected to the aforementioned analysis to explore the specificity of the cell type.

### Relationship to the cytoarchitecture of cortical organization

To explore whether the developmental pattern of the WM connectome aligns with the fundamental cytoarchitecture of cortical organization, we focused on myelin content^43^ and the thicknesses of L1-L6^41^. For each cortical organization map, the vertex values were assigned and averaged to regional values according to the BNA246 template on fsaverage5 space. Then, we calculated Spearman’s correlation coefficient between the developmental slope of the nodal property and the extracted regional values of each cortical organization map. For each laminar thickness, we also calculated a partial correlation coefficient to explore laminar specificity after controlling remaining laminar thickness. The corresponding *p* value was corrected for spatial autocorrelation by calculating the number of times that the correlation coefficients derived from 10,000 spatially constrained null models were greater than the observed correlation coefficient. In addition, FreeSurfer (https://surfer.nmr.mgh.harvard.edu/fswiki/FreeSurferWiki) was used to directly calculate the cortical thickness of each individual and obtain the average cortical thickness for subsequent validation of the cytoarchitecture results.

### Validation analyses

We assessed the consistency of the results with the head movement as an additional covariate. Briefly, we computed the relative mean displacement as the measure of head movement and integrated it into the mixed effect model to delineate the developmental changes of global and nodal properties. The normalized gene weights were derived though PLS correlation between ${\beta }_{{age}}$ of nodal efficiency and gene expression. To assess the consistency of the results, we employed Pearson’s correlation coefficient to compare models with and without the inclusion of head motion. The corresponding *p* value was corrected for spatial autocorrelation by calculating the number of times that the correlation coefficients derived from 10,000 spatially constrained null models were greater than the observed correlation coefficient.

We validated the sensitivity of the result based on a distinct template. A native brain parcellation derived from AAL90^64^ was obtained by applying inverse transformation matrices. For each participant, an FA$\times$FN-weighted WM network was constructed. Statistical analyses were utilized for mainly global and nodal network properties to investigate the effects of age and sex differences on the topological organization of the AAL90 WM network.

We also proceeded to create networks weighted by FN, FA, and 1/MD employing the BNA246 template. These networks were formulated to assess the results of diverse connection-weighted approaches, complementing the principal findings of global and nodal properties. Spearman’s correlation coefficients were computed to quantify the association between the developmental slopes of node properties across distinct weighted networks. The corresponding *p* value was corrected for spatial autocorrelation by determining the frequency with which correlation coefficients from 10,000 spatially constrained null models exceeded the observed correlation coefficient. To evaluate the robustness of the tractography approach, following our previous methodological evaluation study^107^, an FA$\times$FN-weighted network derived by the Camino toolbox (http://camino.cs.ucl.ac.uk/) and a probabilistic tractography weighted network^119^ were constructed based on a ball-and-stick model estimated from bedpostx results^65^. The gene association and cell type-specific analysis were performed as for the nodal efficiency ${\beta }_{{age}}$.

We employed the ABAnnotate toolbox^66,67^, which takes into account both gene coexpression and spatial autocorrelation, to validate the enrichment results of spatial correlation between the developmental slope ${\beta }_{{age}}$ of WM nodal efficiency and gene expression profiles. Specially, we conducted gene category enrichment analysis on GO categories for positively and negatively correlated genes selected by PLS, where the weight of genes served as the gene score. Then, we generated 10,000 spatially autocorrelated maps^57^ of the developmental ${\beta }_{{age}}$ to estimate a category-level null distribution of gene score. Finally, we inferred the statistical *p* value on category enrichment by comparing observed mean gene score to the null distribution. Multiple comparison correction was applied using the FDR method.

To evaluate whether the heterogeneous spatial development and transcriptomic architecture obtained from our study were replicated in another independent cohort, the HCP-D 2.0 data release was utilized for validation. We applied minimal preprocessing pipelines^120^ according to imaging acquisition details^45,46^. Following our previous methodological evaluation study^107^, the Camino toolbox (http://camino.cs.ucl.ac.uk/) was used to reconstruct fibers with a ball-and-stick model estimated from bedpostx results^65^ and to generate an FA$\times$FN-weighted network with the BNA246 template. After demographic matching and quality control during MRI preprocessing, we selected a cross-sectional cohort composed of 179 typically developing children (age range of 6–13 years, 76 males and 103 females) who were unrelated to others in the HCP-D (Supplementary Table 2). The network properties were calculated by GRETNA^121^ and the general linear model was applied to analyse age-related changes. The subsequent gene association analysis, cell type-specific analysis and cortical organization correlation were adopted for the CBD cohort. Pearson’s correlation coefficient was applied to calculate the consistency of nodal efficiency ${\beta }_{{age}}$ between two cohorts and to measure the consistency of normalized gene weights between two cohorts. The corresponding *p* value was corrected for spatial autocorrelation by calculating the number of times that the correlation coefficients derived from 10,000 spatially constrained null models were greater than the observed correlation coefficient.

### Statistics and reproducibility

Mixed effect model^52^ was performed to obtain the statistical correlation between WM network properties and age (*n* = 1033). For linear associations between the spatial development slopes of WM nodal efficiency and other brain phenotypes (gene expression profiles and cortical organization maps), we generated 10,000 surrogate maps of development slopes^57^ to correct for spatial autocorrelation of MRI data. All linear models were fitted for the original data as well as 10,000 corresponding surrogate maps. P-values were obtained by the occupied null models (<5th, or >95th centile). Six analysis strategies were considered to verify the reproducibility, including (i) head movement as an additional covariate (*n* = 1033); (ii) defining brain nodes based on a distinct brain template (*n* = 1033); (iii) using different connection-weighted approaches (*n* = 1033); (iv) using another tractography approach (*n* = 1033); (v) using another gene category enrichment analysis pipeline; and (vi) using another independent dataset (HCP-D, *n* = 179). Spatial development patterns of WM network properties, gene association, and cell type-specific analysis were examined in these cases.

### Reporting summary

Further information on research design is available in the Nature Portfolio Reporting Summary linked to this article.

### Supplementary information

Supplementary Information

Reporting summary

## Data Availability

The CBD data that support the findings of this study are available from the corresponding author upon reasonable request. The HCP-D 2.0 release data that support the findings of this study are publicly available on https://www.humanconnectome.org/study/hcp-lifespan-development. The AHBA dataset are available on the Allen Brain Atlas (https://human.brain-map.org/static/download). The processed transcriptomic data in this study are available from the corresponding author upon reasonable request. The source data underlying Figs. 1, 3, 4, 5, and 6 can be accessed at https://figshare.com/articles/dataset/WM-connectome-development/24588585.

## Code availability

The code for preprocessing of the AHBA dataset can be found at https://github.com/BMHLab/AHBAprocessing. The gene enrichment analysis is performed at https://metascape.org/gp/index.html#/main/step1. The spatial permutation testing is performed based on BrainSpace (https://brainspace.readthedocs.io/en/latest/index.html). The minimal preprocessing pipelines can be found at https://github.com/Washington-University/HCPpipelines. The code relevant to this study can be accessed at https://github.com/FelixFengCN/WM-connectome-development^122^.
