# Author Correction: Longitudinal development of the human white matter structural connectome and its association with brain transcriptomic and cellular architecture

**DOI:** 10.1038/s42003-024-05771-z

**Published:** 2024-01-11

**Authors:** Guozheng Feng, Rui Chen, Rui Zhao, Yuanyuan Li, Leilei Ma, Yanpei Wang, Weiwei Men, Jiahong Gao, Shuping Tan, Jian Cheng, Yong He, Shaozheng Qin, Qi Dong, Sha Tao, Ni Shu

**Affiliations:** 1 State Key Laboratory of Cognitive Neuroscience and Learning & IDG/McGovern Institute for Brain Research, Beijing Normal University, Beijing, China; 2 BABRI Centre, Beijing Normal University, Beijing, China; 3 Beijing Key Laboratory of Brain Imaging and Connectomics, Beijing Normal University, Beijing, China; 4 College of Life Sciences, Beijing Normal University, Beijing, China; 5 Beijing Key Laboratory of Gene Resource and Molecular Development, Beijing, China; 6 Center for MRI Research, Academy for Advanced Interdisciplinary Studies, Peking University, Beijing, China; 7 Beijing Huilongguan Hospital, Peking University Huilongguan Clinical Medical School, Beijing, China; 8 School of Computer Science and Engineering, Beihang University, Beijing, China

**Keywords:** Development of the nervous system, Brain, Network models

Correction to: *Communications Biology* 10.1038/s42003-023-05647-8, published online 12 December 2023.

In this article the affiliation details for Yong He were incorrectly given as ‘Yong He^1,2,3^’ but should have been ‘Yong He^1,3^’. The original article has been corrected.

