# Supplementary material for: Longitudinal development of the human white matter structural connectome and its association with brain transcriptomic and cellular architecture
Source: Commun Biol. 2023 Dec 12;6:1257. doi: 10.1038/s42003-023-05647-8 (PMC10716168; doi:10.1038/s42003-023-05647-8)
Supplement: Supplementary file 1 — Supplementary Information [file 42003_2023_5647_MOESM1_ESM.pdf]

Supplementary Information for

Longitudinal development of the human white matter structural connectome and its association with brain transcriptomic and cellular architecture

Guozheng Feng, Rui Chen, Rui Zhao, Yuanyuan Li, Leilei Ma, Yanpei Wang, Weiwei Men, Jiahong Gao, Shuping Tan, Jian Cheng, Yong He, Shaozheng Qin, Qi Dong, Sha Tao*, Ni Shu*

***Corresponding To:** Sha Tao, PhD, State Key Laboratory of Cognitive Neuroscience and Learning & IDG/McGovern Institute for Brain Research, Beijing Normal University, Beijing 100875, China; Tel: +86 10 58808778; Fax: +86 10 58802048; E-mail: taosha@bnu.edu.cn.

Ni Shu, PhD, State Key Laboratory of Cognitive Neuroscience and Learning & IDG/McGovern Institute for Brain Research, Beijing Normal University, Beijing 100875, China; Tel: +86 10 58806154; Fax: +86 10 58806154; E-mail: nshu@bnu.edu.cn.

Content:

- **Supplementary Table 1.** The demography of discovery cohort (CBD).
- **Supplementary Table 2.** The demography of validation cohort (HCP-D).
- **Supplementary Table 3.** Age-related changes in global properties of FN network.
- **Supplementary Table 4.** Age-related changes in global properties of

FA network.

- **Supplementary Table 5.** Age-related changes in global properties of 1/MD network.
- **Supplementary Table 6.** Age-related changes in global properties of ball-and-stick model-derived FN×FA network.
- **Supplementary Table 7.** Correlation of developmental slope of nodal properties across different weighted networks.
- **Supplementary Table 8.** Age-related changes of global properties in the independent validation set (HCP-D).
- **Supplementary Figure 1.** Longitudinal changes of global properties during childhood and adolescence.
- **Supplementary Figure 2.** Developmental alterations of slice-by-slice profiles on nodal properties.
- **Supplementary Figure 3.** Longitudinal alterations of various types of connection during childhood and adolescence.
- **Supplementary Figure 4.** Association between the developmental alteration of nodal efficiency and cortical thickness in CBD cohort.
- **Supplementary Figure 5.** Reproducible development alterations considering the head movement as an additional covariate.
- **Supplementary Figure 6.** Reproducible development alterations with the AAL90 parcellation scheme.
- **Supplementary Figure 7.** The results of development alterations

with different connection-weighted and tractography schemes.

- **Supplementary Figure 8.** Gene transcriptional correlation and cell type-specific analysis with the ball-and-stick model-derived FN×FA network.
- **Supplementary Figure 9.** Gene transcriptional correlation and cell type-specific analysis with the probabilistic tractography network.
- **Supplementary Figure 10.** Gene category enrichment analysis with ABAnnotate toolbox.
- **Supplementary Figure 11.** Reproducible validation in the independent validation cohort (HCP-D).

Supplementary Tables

Supplementary Table 1. The demographic characteristics of the discovery cohort (CBD).

Age		[6,7)	[7,8)	[8,9)	[9,10)	[10,11)	[11,12)	[12,13]	total
Centre1	1 time	13/19	39/38	73/47	65/53	40/31	22/22	7/1	470
	2 times	1/0	11/11	18/16	41/32	33/27	20/13	7/11	241
	3 times	0/0	0/0	3/4	11/12	25/12	9/11	8/7	112
Centre2	1 time	5/1	16/6	19/12	18/14	18/11	3/6	1/4	134
	2 times	0/0	3/1	5/4	12/4	7/7	13/7	1/3	67
	3 times	0/0	0/0	0/0	0/0	0/1	2/0	4/2	9
Total		39	125	201	262	222	128	56	1033

Supplementary Table 2. The demographic of the validation cohort (HCP-D).

Age	[6,7)	[7,8)	[8,9)	[9,10)	[10,11)	[11,12)	[12,13]	Total
Male	1	3	13	13	21	11	14	76
Female	4	5	15	31	17	12	19	103
Total	5	8	28	44	38	23	33	179

Supplementary Table 3. Age-related changes in global properties of FN network.

	Aged β	CI	t	p
Global efficiency	0.68	[0.59,0.78]	14.46	8.29E-43
Local efficiency	1.00	[0.85,1.17]	12.73	2.07E-34
Shortest path	-1.60E-03	[-1.82E-03,-1.37E-03]	-13.87	7.22E-40
Network strength	1.75E+01	[1.55E+01,1.96E+01]	16.96	1.96E-55
Clustering coefficient	-1.15E-05	[-1.38E-04,1.15E-04]	-0.18	0.86
Small-World (γ)	-6.75E-02	[-9.69E-02,-3.81E-02]	-4.55	6.04E-06
Small-World (λ)	-1.20E-03	[-3.16E-03,7.95E-04]	-1.18	0.24
Small-World (σ)	-5.41E-02	[-7.81E-02,-3.01E-02]	-4.44	1.02E-05

Supplementary Table 4. Age-related changes in global properties of FA network.

	Aged β	CI	t	p
Global efficiency	2.50E-03	[2.23E-03,2.81E-03]	17.09	1.16E-56
Local efficiency	3.5E-03	[3.08E-03,3.96E-03]	15.75	5.06E-50
Shortest path	-1.11E-01	[-1.23E-01,-9.81E-02]	-16.98	1.38E-56
Network strength	1.14E-01	[1.00E-01,1.27E-01]	16.59	6.78E-54
Clustering coefficient	6.16E-04	[1.01E-04,1.13E-03]	2.35	0.02
Small-World (γ)	-8.90E-02	[-1.11E-01,-6.65E-02]	-7.82	1.32E-14
Small-World (λ)	-1.90E-03	[-2.51E-03,-1.34E-03]	-6.45	1.75E-10
Small-World (σ)	-6.82E-02	[-8.67E-02,-4.97E-02]	-7.30	6.00E-13

Supplementary Table 5. Age-related changes in global properties of 1/MD network.

	Aged β	CI	t	p
Global efficiency	6.62	[5.96,7.27]	19.84	4.41E-72
Local efficiency	9.96	[8.78,11.13]	16.68	3.02E-55
Shortest path	-3.18E-05	[-3.50E-05,-2.86E-05]	-19.70	3.48E-72
Network strength	3.31E+02	[2.95 E+02,3.68 E+02]	17.87	8.66E-61
Clustering coefficient	1.00E-03	[-8.50E-04,2.90E-03]	1.07	0.28
Small-World (γ)	-8.82E-02	[-1.11E-01,-6.56E-02]	-7.70	3.27E-14
Small-World (λ)	-2.50E-03	[-3.07E-03,-1.91E-03]	-8.48	7.65E-17
Small-World (σ)	-6.32E-02	[-8.11E-02,-4.54E-02]	-7.03	4.02E-12

Supplementary Table 6. Age-related changes in global properties of ball-and-stick model-derived FN×FA network.

	Aged β	CI	t	p
Global efficiency	5.38E-02	[4.43E-02,6.34E-02]	11.17	2.87E-27
Local efficiency	6.94E-02	[5.64E-02,8.25E-02]	10.49	2.06E-24
Shortest path	-7.00E-03	[-8.26E-03,-5.75E-03]	-11.01	1.20E-26
Network strength	1.72	[1.47,1.97]	13.63	1.39E-38
Clustering coefficient	-1.09E-04	[-1.00E-04,2.58E-04]	-1.70	0.09
Small-World (γ)	-1.93E-02	[-2.99E-02,-8.88E-03]	-3.65	2.81E-04
Small-World (λ)	2.43E-03	[1.01E-03,3.86E-03]	3.39	7.29E-04
Small-World (σ)	-5.51E-02	[-3.35E-02,-1.66E-02]	-5.82	7.94E-09

Supplementary Table 7. Correlation of developmental slope of nodal properties across different weighted networks.

		FN	FA	1/MD	FN×FA[a]
Nodal efficiency	FN×FA	0.97***	0.47***	0.49***	0.57***
	FN		0.40**	0.47***	0.56***
	FA			0.91***	0.55***
	1/MD				0.55***
Local efficiency	FN×FA	0.98***	0.57***	0.56***	0.57***
	FN		0.55***	0.58***	0.56***
	FA			0.87***	0.54***
	1/MD				0.54***
Degree centrality	FN×FA	0.98***	0.57***	0.57***	0.61***
	FN		0.58***	0.60***	0.58***
	FA			0.97***	0.61***
	1/MD				0.59***

Of note, FN×FA[a]: ball-and-stick model-derived FN×FA network; ***: $p<0.001$; **: $p<0.01$; *: $p<0.05$, permutation test with SAC.

Supplementary Table 8. Age-related changes in global properties in the independent validation set (HCP-D).

	Aged β	CI	t	p
Global efficiency	0.14	[0.05,0.23]	3.07	2.51E-03
Local efficiency	0.16	[0.05,0.27]	2.76	6.44E-03
Shortest path	-8.13E-03	[-1.41E-02,-2.11E-03]	-2.67	8.38E-03
Network strength	4.62	[2.21,7.03]	3.78	2.13E-04
Clustering coefficient	-9.71E-05	[-2.28E-04,-3.39E-05]	-1.46	0.15
Small-World (γ)	-5.37E-02	[-8.06E-02,-2.68E-02]	-3.94	1.18E-04
Small-World (λ)	3.93E-03	[-8.18E-04,8.67E-03]	1.63	0.10
Small-World (σ)	-5.55E-02	[-7.81E-02,-3.28E-02]	-4.83	3.03E-06

Supplementary Figures

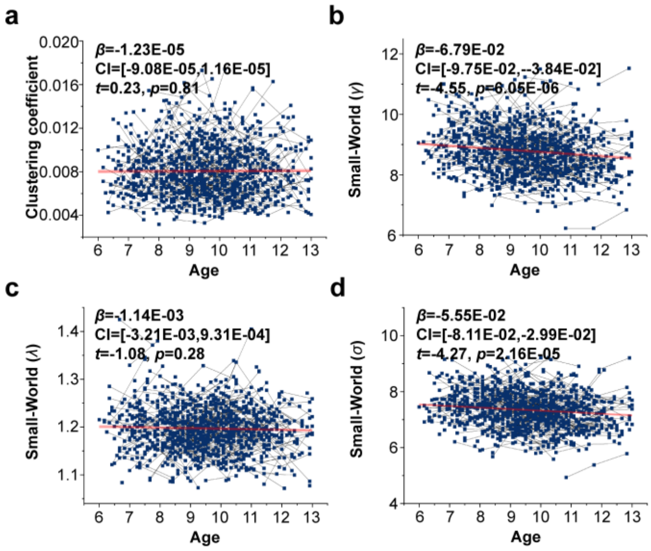

Supplementary Figure 1. Longitudinal changes in global properties during childhood and adolescence (*n* = 1033 scans). a Clustering coefficient. **b-d** Small-world properties.

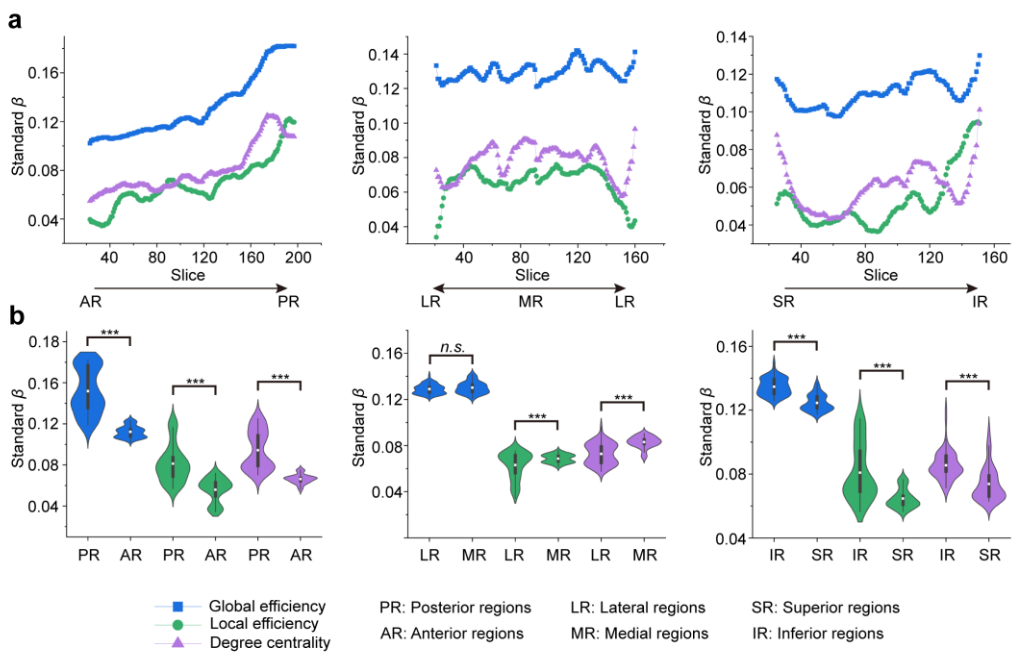

Supplementary Figure 2. Developmental alterations of slice-by-slice profiles on nodal network properties. a Standard development slope of nodal property changes along anterior to posterior, medial to lateral and superior to inferior directions. **b** Differences between the development slopes of slices belonging to the anterior and posterior, medial and lateral and superior and inferior planes. In each violin plots, the box plots show the median and interquartile range, the square point depicts mean value, and the whiskers depict range of outlier with coefficient = 1.5. *n.s.*: p>0.05; ***: p<0.001, t-test.

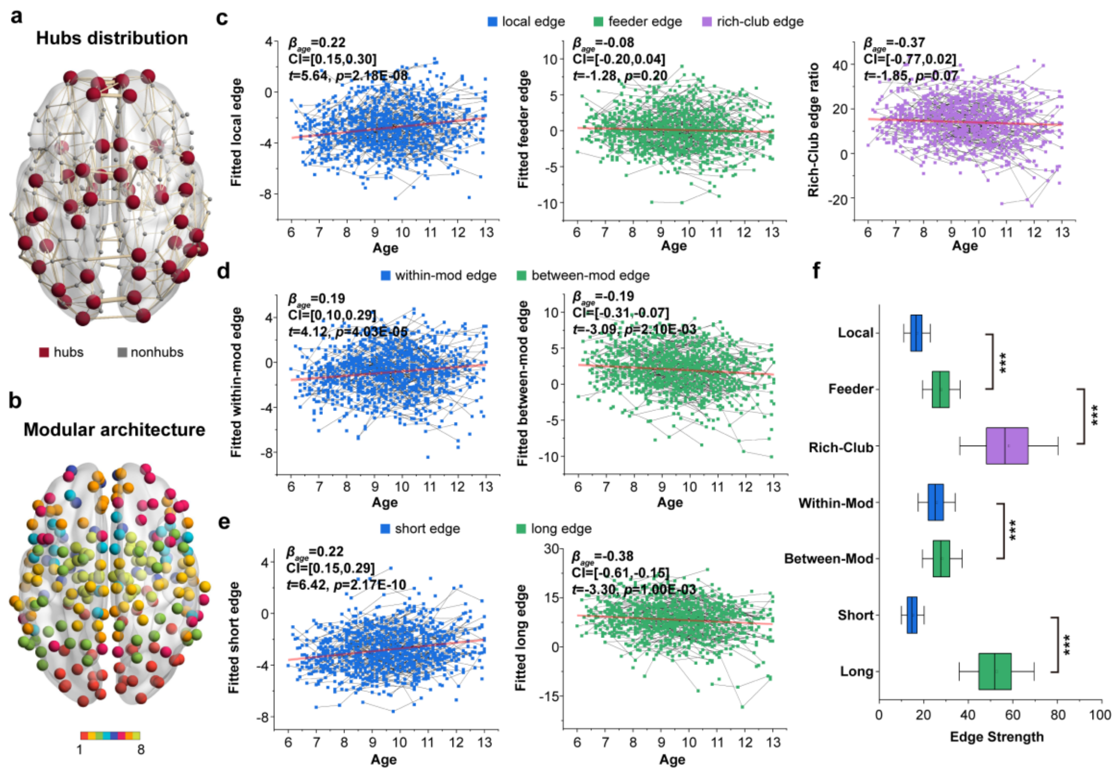

Supplementary Figure 3. Longitudinal alteration in various types of connections during childhood and adolescence. a Hub distribution of the group-averaged WM connectome with the hub nodes in red and nonhub nodes in grey, local edge in fine line, feeder edge in medium line, and rich-club edge in rough line. **b** Modular architecture according to Yeo's brain parcellation, and different colours correspond to different modules. **c** Developmental changes in edge ratio based on hub distribution type (n = 1033 scans). **d** Developmental changes in the edge ratio based on modular architecture type (n = 1033 scans). **e** Developmental changes in the edge ratio of short and long edges (n = 1033 scans). **f** Edge strength of various edges and pairwise comparisons of edge strength within the same typing strategy (n = 1033 scans). In **f**, the boxes show the median and interquartile range, the square point depicts mean value, and the whiskers depict 1.5× standard deviation. ***: p<0.001, t-test.

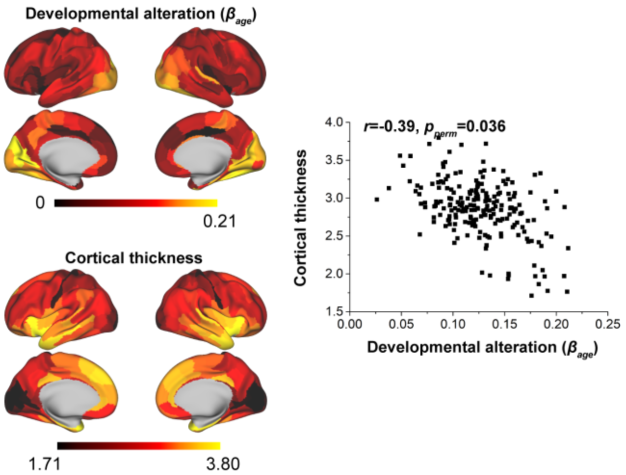

Supplementary Figure 4. Association between the developmental alteration of nodal efficiency and cortical thickness in CBD cohort.

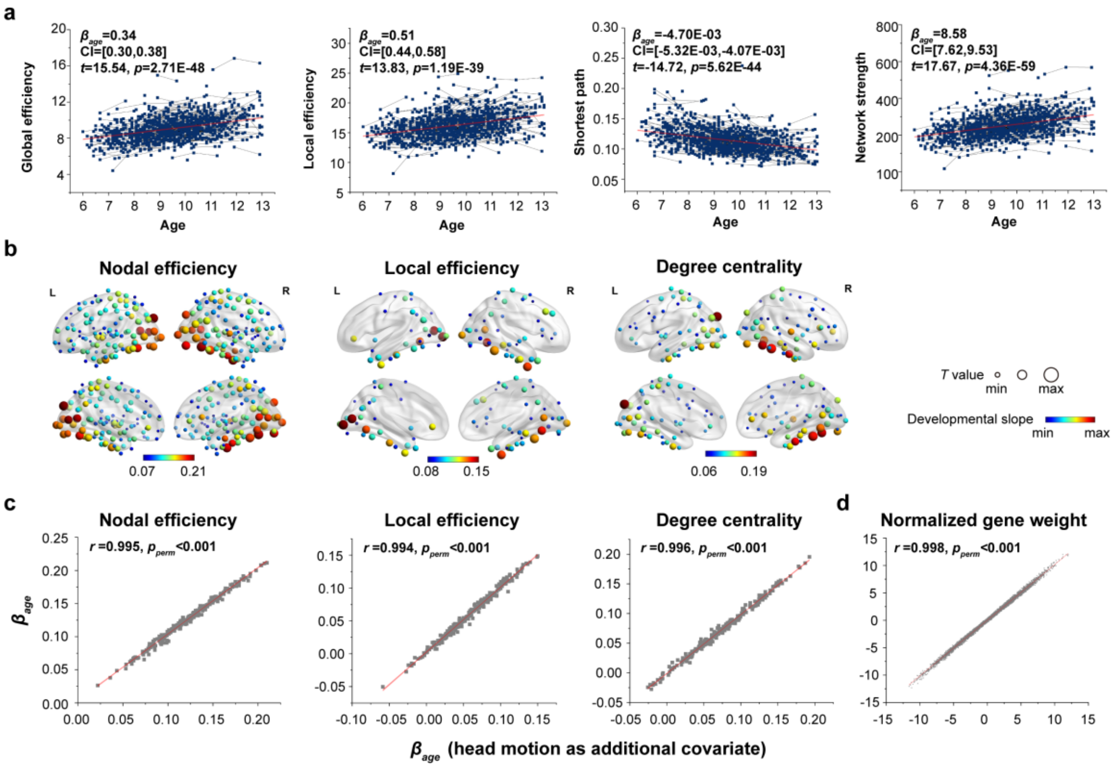

Supplementary Figure 5. Reproducible development alterations considering the head movement as an additional covariate. a Age effect on mainly global network properties (n = 1033 scans). **b** Spatial patterns with significant development (p<0.05, Bonferroni correction, n = 1033 scans) in various nodal properties. The size of the circle was proportional to the statistical t value, and its colour indicated the developmental slope (standard effect value β_{age}), with red for large changes and blue for small changes. **c** Consistency of development slope β_{age} in nodal property with and without head movement. **d** Consistency of normalized gene weight with and without head movement.

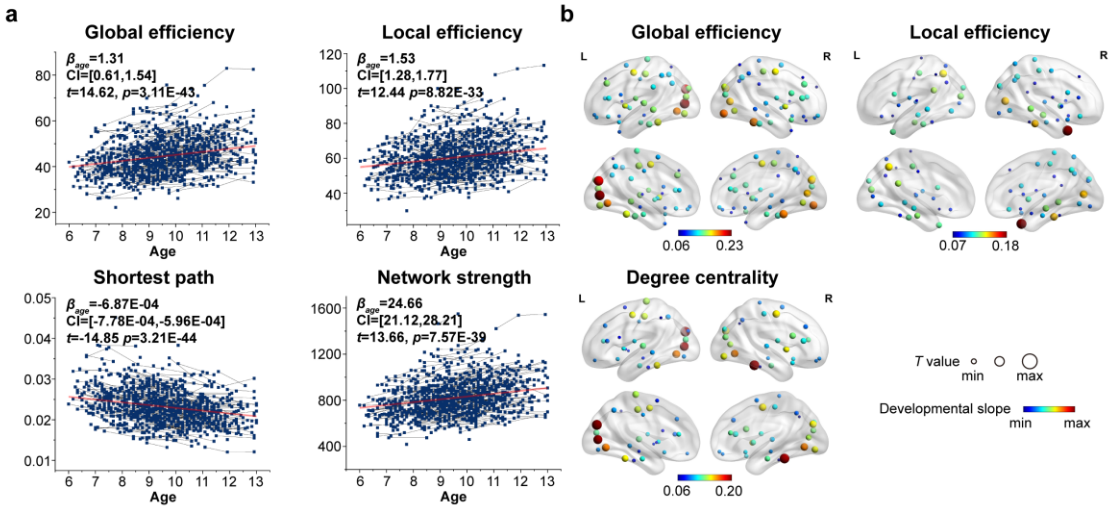

Supplementary Figure 6. Reproducible development alterations with the AAL90 parcellation scheme. a Standardized age effect on four global network properties (*n* = 1033 scans). **b** Spatial distribution of brain regions with significant development (p<0.05, Bonferroni correction, *n* = 1033 scans) in three nodal properties. The size of the circle node indicates the statistical t value, and its colour denotes the developmental slope (standard effect value β_{age}), with red for large changes and blue for small changes.

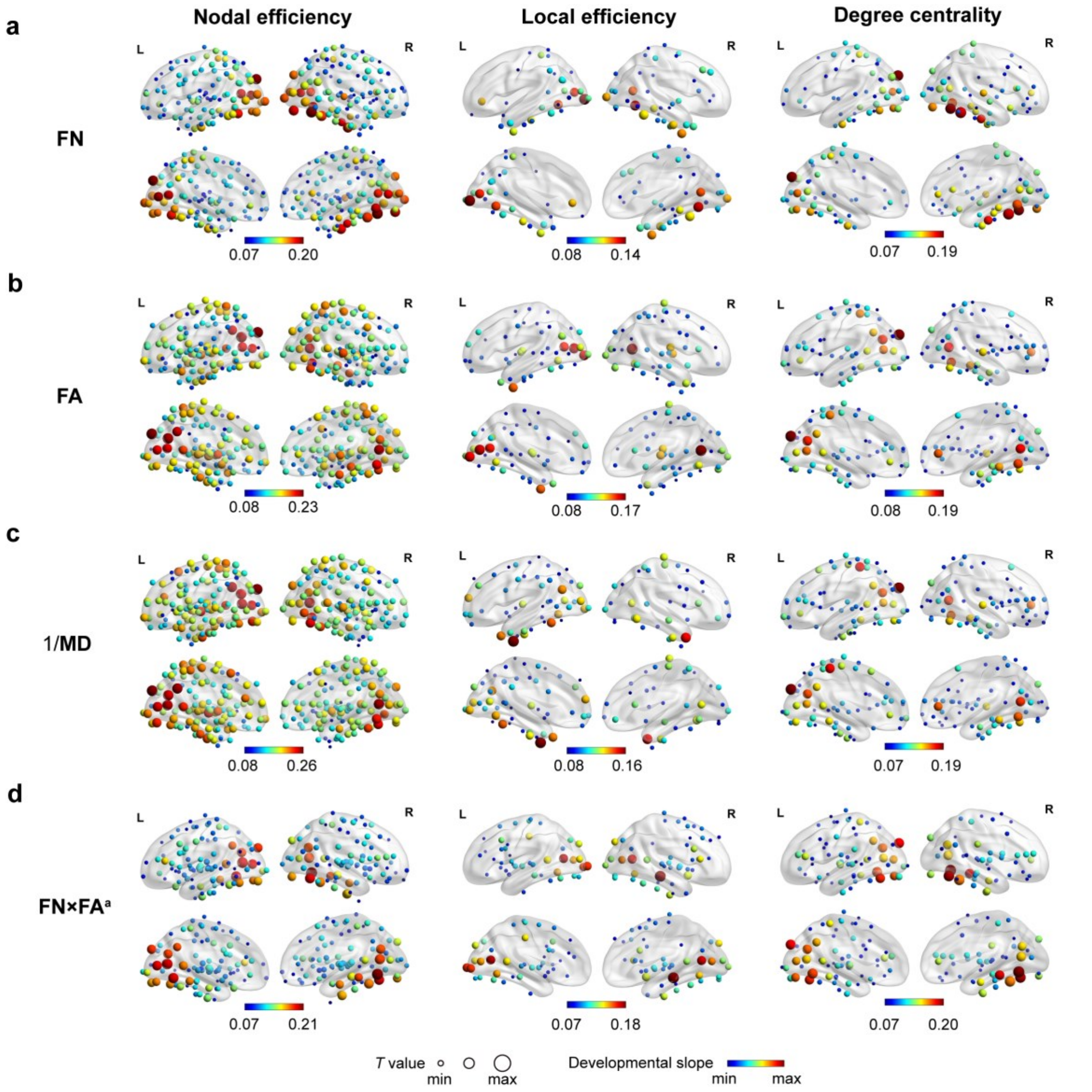

Supplementary Figure 7. The results of development alterations with different connection-weighted and tractography schemes. Spatial distribution of brain regions with significant development (p<0.05, Bonferroni correction, n = 1033 scans) in FN (**a**), FA (**b**), 1/MD (**c**), and FN ×FA[a] (**d**). The size of the circle node indicates the statistical t value, and its colour denotes the developmental slope (standard effect value β_{age}), with red for large changes and blue for small changes. Of note, FN×FA[a]: ball-and-stick model-derived FN×FA network.

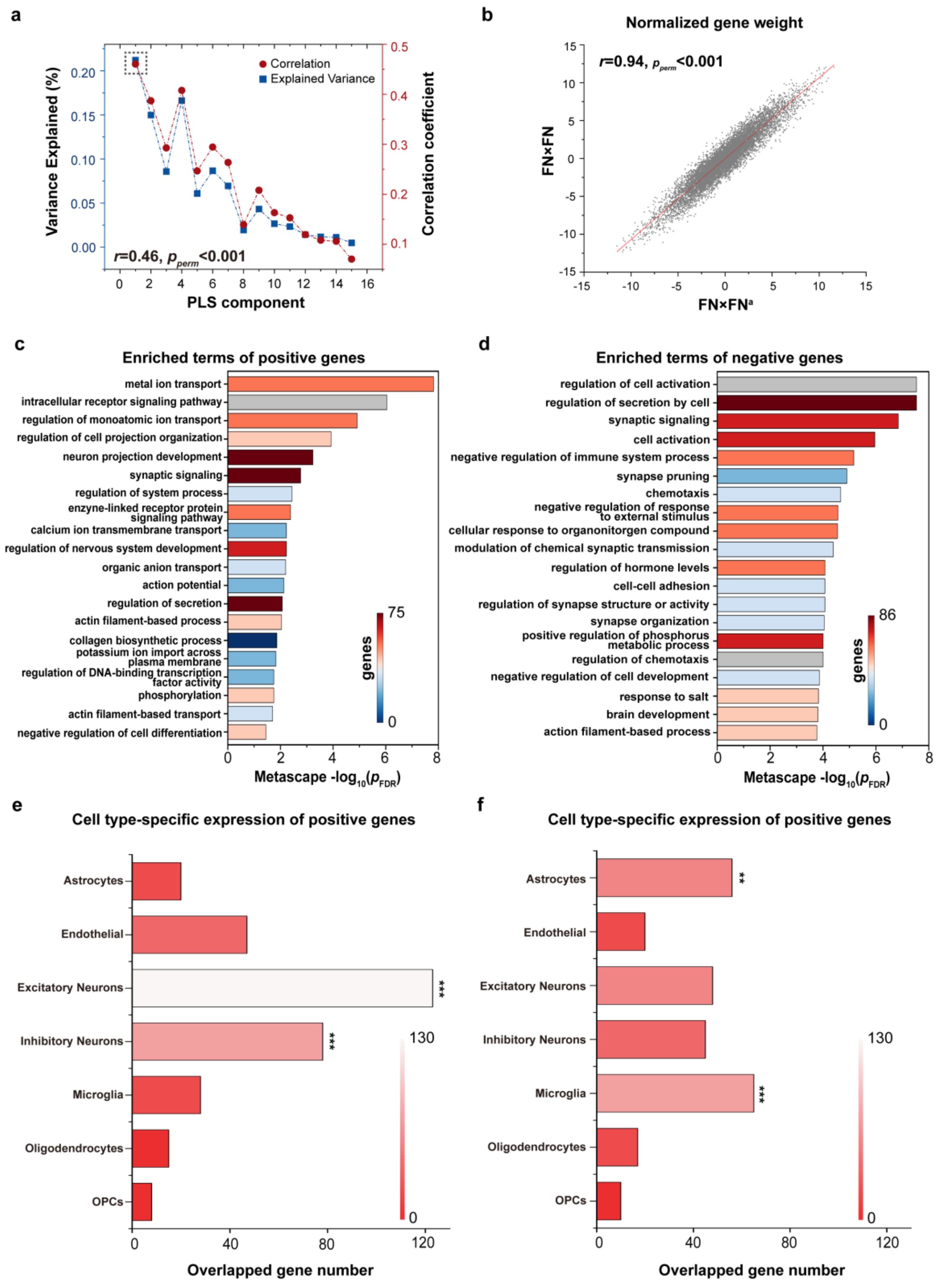

Supplementary Figure 8. Gene transcriptional correlation and cell type-specific analysis with the ball-and-stick model-derived FN×FA network. a Explained ratios (left vertical axis) and correlation coefficients (right vertical axis) for the first 15 components obtained from the PLS regression analysis. **b** Consistency of normalized gene weight between the single-tensor model-derived FN×FA network and the ball-and-stick model-derived FN×FA network. Enriched terms of

positive genes (**c**) and negative genes (**d**). The length of the bar denotes the enrichment significance and its colour denotes the number of input genes falling under that term. Cell type-specific expression of positive genes (**e**) and negative genes (**f**). The length and colour of the bar shows overlapping numbers of the selected genes in each cell class. Of note, OPCs: oligodendrocyte precursors. **: $p<0.01$; ***: $p<0.001$, permutation test.

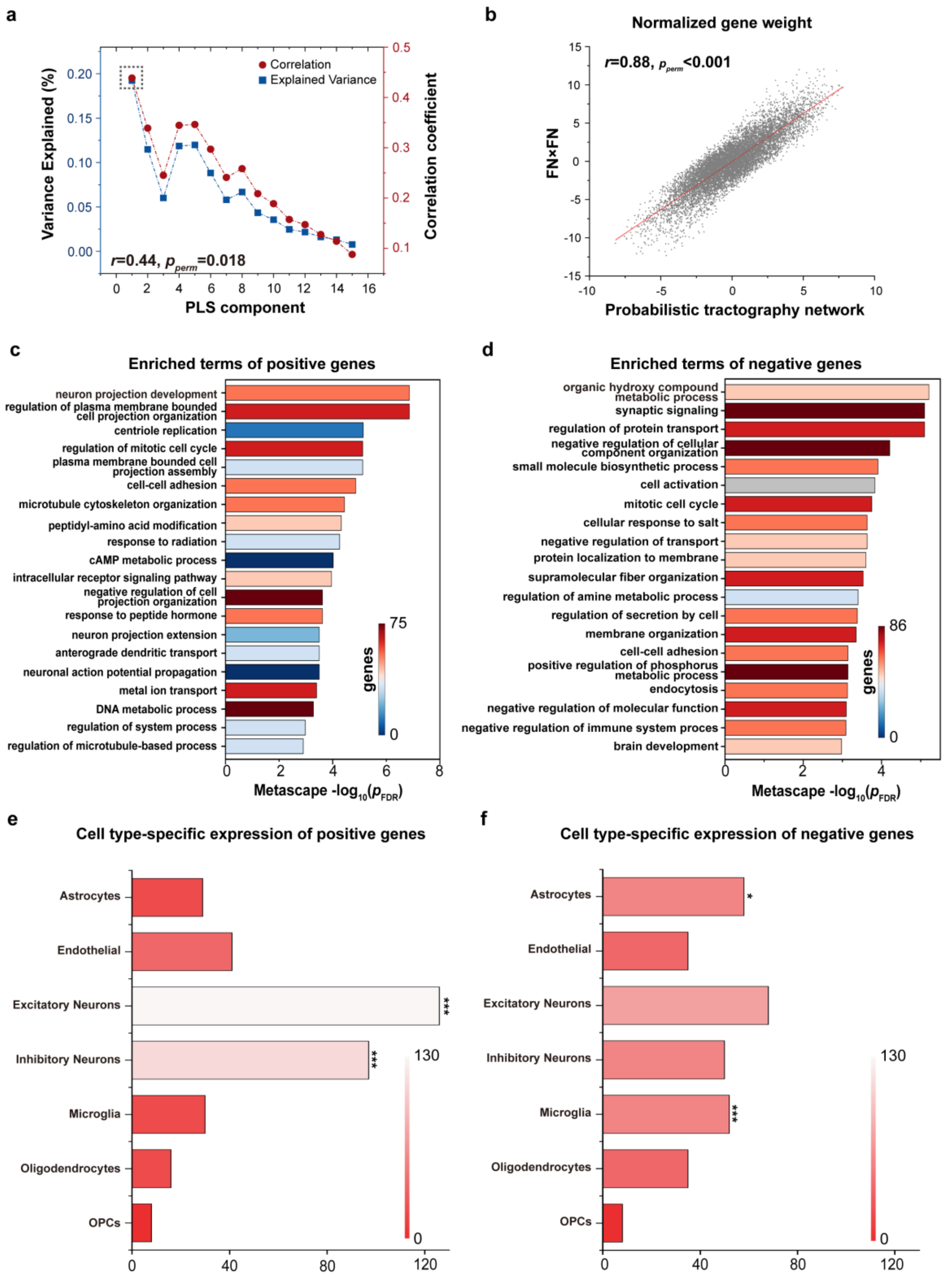

Supplementary Figure 9. Gene transcriptional correlation and cell type-specific analysis with the probabilistic tractography network. a Explained ratios (left vertical axis) and correlation coefficients (right vertical axis) for the first 15 components obtained from the PLS regression analysis. **b** Consistency of normalized gene weight between the single-tensor model-derived FN× FA network and the probabilistic tractography network. Enriched terms of positive genes (**c**) and

negative genes (**d**). The length of the bar denotes the enrichment significance and its colour denotes the number of input genes falling under that term. Cell type-specific expression of positive genes (**e**) and negative genes (**f**). The length and colour of the bar shows overlapping numbers of the selected genes in each cell class. Of note, OPCs: oligodendrocyte precursors. *: $p<0.05$; ***: $p<0.001$, permutation test.

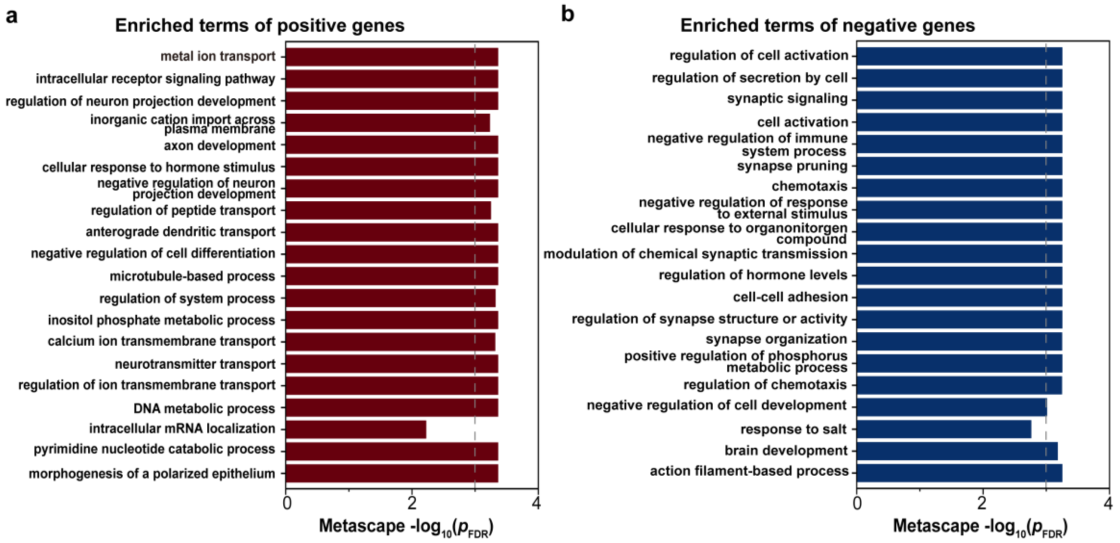

Supplementary Figure 10. Gene category enrichment analysis with ABAnnotate toolbox. a Enriched terms of positive genes. **b** Enriched terms of negative genes.

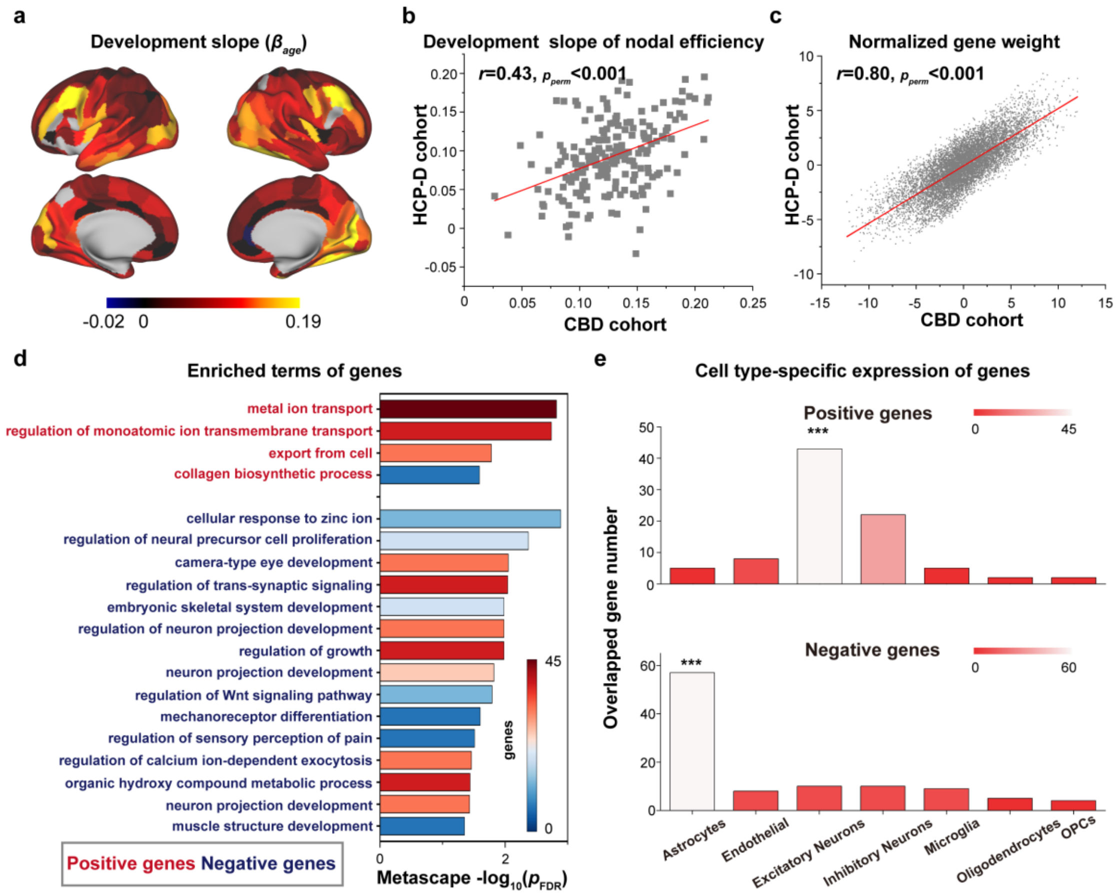

Supplementary Figure 11. Reproducible validation in the independent validation set (HCP-D). **a** The map of standardized development slope (β_{age}) in nodal efficiency across 199 brain regions. **b** Consistency of development slope in nodal efficiency between the CBD cohort and the HCP-D cohort. **c** Consistency of normalized gene weight between the CBD cohort and the HCP-D cohort. **d** Typically enriched terms within each cluster. The length of the bar denotes enrichment significance, and its colour denotes the number of input genes falling under that term. **e** Overlapping numbers of the selected genes in each cell class. Of note, OPCs: oligodendrocyte precursors. ***: p<0.001, permutation test.
