# Supplementary material for: Longitudinal development of the human white matter structural connectome and its association with brain transcriptomic and cellular architecture
Source: Commun Biol. 2023 Dec 12;6:1257. doi: 10.1038/s42003-023-05647-8 (PMC10716168; doi:10.1038/s42003-023-05647-8)
Supplement: Supplementary file 2 — Reporting summary [file 42003_2023_5647_MOESM2_ESM.pdf]

nature portfolio

Corresponding author(s): DBPR COMMSBIO-23-1233B

Last updated by author(s): Nov 21, 2023

Reporting Summary

Nature Portfolio wishes to improve the reproducibility of the work that we publish. This form provides structure for consistency and transparency in reporting. For further information on Nature Portfolio policies, see our Editorial Policies and the Editorial Policy Checklist.

Statistics

For all statistical analyses, confirm that the following items are present in the figure legend, table legend, main text, or Methods section.

n/a	Confirmed	
☐	☒	The exact sample size (n) for each experimental group/condition, given as a discrete number and unit of measurement
☐	☒	A statement on whether measurements were taken from distinct samples or whether the same sample was measured repeatedly
☐	☒	The statistical test(s) used AND whether they are one- or two-sided<br>*Only common tests should be described solely by name; describe more complex techniques in the Methods section.*
☐	☒	A description of all covariates tested
☐	☒	A description of any assumptions or corrections, such as tests of normality and adjustment for multiple comparisons
☐	☒	A full description of the statistical parameters including central tendency (e.g. means) or other basic estimates (e.g. regression coefficient) AND variation (e.g. standard deviation) or associated estimates of uncertainty (e.g. confidence intervals)
☐	☒	For null hypothesis testing, the test statistic (e.g. F, t, r) with confidence intervals, effect sizes, degrees of freedom and P value noted<br>*Give P values as exact values whenever suitable.*
☒	☐	For Bayesian analysis, information on the choice of priors and Markov chain Monte Carlo settings
☒	☐	For hierarchical and complex designs, identification of the appropriate level for tests and full reporting of outcomes
☐	☒	Estimates of effect sizes (e.g. Cohen's d, Pearson's r), indicating how they were calculated

Our web collection on statistics for biologists contains articles on many of the points above.

Software and code

Policy information about availability of computer code

Data collection	No software was used in the data collection process.
Data analysis	The code for preprocessing of the AHBA dataset can be found at https://github.com/BMHLab/AHBAprocessing. The gene enrichment analysis is performed at https://metascape.org/gp/index.html#/main/step1. The spatial permutation testing is performed based on BrainSpace (https://brainspace.readthedocs.io/en/latest/index.html). The minimal preprocessing pipelines can be found at https://github.com/Washington-University/HCPpipelines. The code relevant to this study can be accessed at https://github.com/FelixFengCN/WM-connectome-development.

For manuscripts utilizing custom algorithms or software that are central to the research but not yet described in published literature, software must be made available to editors and reviewers. We strongly encourage code deposition in a community repository (e.g. GitHub). See the Nature Portfolio guidelines for submitting code & software for further information.

Data

Policy information about availability of data

All manuscripts must include a data availability statement. This statement should provide the following information, where applicable:

- Accession codes, unique identifiers, or web links for publicly available datasets
- A description of any restrictions on data availability
- For clinical datasets or third party data, please ensure that the statement adheres to our policy

The CBD data that support the findings of this study are available from the corresponding author upon reasonable request. The HCP-D 2.0 release data that support the findings of this study are publicly available on https://www.humanconnectome.org/study/hcp-lifespan-development. The AHBA dataset are available on the Allen Brain Atlas (https://human.brain-map.org/static/download). The source data underlying Figs. 2, 3, 4, 5, and 6 can be accessed at https://figshare.com/articles/dataset/WM-connectome-development/24588585.

Research involving human participants, their data, or biological material

Policy information about studies with human participants or human data. See also policy information about sex, gender (identity/presentation), and sexual orientation and race, ethnicity and racism.

Reporting on sex and gender	CBD cohort includes 604 typically-developing children (339 males and 266 females).HCP-D includes 179 typically-developing children (76 males and 103 females). In this study, we exclusively considered biological sex in our analyses. Sex information was obtained through reports provided by guardians. No significant findings or outcomes related to sex were identified.
Reporting on race, ethnicity, or other socially relevant groupings	No socially constructed or socially relevant categorical variables were used in this study.
Population characteristics	There were no covariate population characteristics included in the study.
Recruitment	From the CBD cohort, typically developing children were recruited from Beijing primary schools. The exclusion criteria included the presence of intellectual or developmental abnormalities, a history of neurological or psychiatric disorders, the use of psychoactive drugs and the presence of a significant head injury.
Ethics oversight	This study was conducted according to the guidelines of the Declaration of Helsinki and was approved by Beijing Normal University Institutional Review Board.

Note that full information on the approval of the study protocol must also be provided in the manuscript.

Field-specific reporting

Please select the one below that is the best fit for your research. If you are not sure, read the appropriate sections before making your selection.

☒ Life sciences ☐ Behavioural & social sciences ☐ Ecological, evolutionary & environmental sciences

For a reference copy of the document with all sections, see nature.com/documents/nr-reporting-summary-flat.pdf

Life sciences study design

All studies must disclose on these points even when the disclosure is negative.

Sample size	No sample-size calculation was performed. The sample size of this study is larger than that of other similar studies. This supports the validity of our findings.
Data exclusions	Out of the original 1072 T1 images, 32 were excluded due to poor image quality. Out of the 1053 dMRI images initially acquired, 1033 images passed the quality control. Finally, 1033 scans with both T1 and dMRI images were included in the subsequent analysis.
Replication	Six analysis strategies were considered to verify the reproducibility, including (i) head movement as an additional covariate (n = 1033); (ii) defining brain nodes based on a distinct brain template (n = 1033); (iii) using different connection-weighted approaches (n = 1033); (iv) using another tractography approach (n = 1033); (v) using another gene category enrichment analysis pipeline; and (vi) using another independent dataset (HCP-D, n = 179). Spatial development patterns of WM network properties, gene association and cell type-specific analysis were examined in these cases. All attempts at replication were successful, confirming the reproducibility of the experimental findings. The shared documentation and data allowed for external verification, and the consistent results across different replication attempts underscore the reliability of the study outcomes.
Randomization	Randomization was not relevant to our study. Our study did not distinguish or define experimental groups.
Blinding	Blinding was not relevant to our study. Our study did not distinguish or define experimental groups.

Reporting for specific materials, systems and methods

We require information from authors about some types of materials, experimental systems and methods used in many studies. Here, indicate whether each material, system or method listed is relevant to your study. If you are not sure if a list item applies to your research, read the appropriate section before selecting a response.

Materials & experimental systems

n/a	Involved in the study
☒	☐ Antibodies
☒	☐ Eukaryotic cell lines
☒	☐ Palaeontology and archaeology
☒	☐ Animals and other organisms
☒	☐ Clinical data
☒	☐ Dual use research of concern
☒	☐ Plants

Methods

n/a	Involved in the study
☒	☐ ChIP-seq
☒	☐ Flow cytometry
☐	☒ MRI-based neuroimaging

Plants

Seed stocks

Report on the source of all seed stocks or other plant material used. If applicable, state the seed stock centre and catalogue number. If plant specimens were collected from the field, describe the collection location, date and sampling procedures.

Novel plant genotypes

Describe the methods by which all novel plant genotypes were produced. This includes those generated by transgenic approaches, gene editing, chemical/radiation-based mutagenesis and hybridization. For transgenic lines, describe the transformation method, the number of independent lines analyzed and the generation upon which experiments were performed. For gene-edited lines, describe the editor used, the endogenous sequence targeted for editing, the targeting guide RNA sequence (if applicable) and how the editor was applied.

Authentication

Describe any authentication procedures for each seed stock used or novel genotype generated. Describe any experiments used to assess the effect of a mutation and, where applicable, how potential secondary effects (e.g. second site T-DNA insertions, mosiacism, off-target gene editing) were examined.

Magnetic resonance imaging

Experimental design

Design type

Resting state.

Design specifications

We train participants to remain still using mock scanning, offer prizes and praise, keep the participants busy while in the scanner with movies (for the structural and diffusion scans), constrain the head in space with pillows and tape, and conduct the MRI scanning toward the beginning of study visits whenever possible.

Behavioral performance measures

No behavioral performance measures were used in this study.

Acquisition

Imaging type(s)

Structural, Diffusion

Field strength

3.0 T

Sequence & imaging parameters

MRI scanning included the collection of 3D T1-weighted structural MRI with a 1 mm3 isotropic voxel size (TR = 2530 ms, TE = 2.98 ms, TI = 1100 ms, flip angle = 7°, FOV = 256×224 mm2, and 192 sagittal slices) and diffusion-weighted MRI (DWI) with a 2 mm3 isotropic voxel size (64 diffusion directions with b = 1000 s/mm2 and 10 images with b=0 s/mm2, TR = 7500 ms, TE = 64 ms, flip angle = 90°, FOV = 224×224 mm2, and 70 axial slices).

Area of acquisition

whole brain

Diffusion MRI ☒ Used ☐ Not used

Parameters

Diffusion-weighted MRI (DWI) with a 2 mm3 isotropic voxel size (64 diffusion directions with b = 1000 s/mm2 and 10 images with b=0 s/mm2, TR = 7500 ms, TE = 64 ms, flip angle = 90°, FOV = 224×224 mm2, and 70 axial slices).

Preprocessing

Preprocessing software

FMRIB's Diffusion Toolbox of the FMRIB Software Library v6.0.
eddy_correct dti.nii.gz eddyDti.nii.gz 0

```
fslroi eddyDti.nii.gz b0.nii.gz 0 1
bet b0.nii.gz betb0.nii.gz -m -f 0.1
dtifit -k eddyDti.nii.gz -o dtifit -m betb0mask.nii.gz -r bvecs -b bvals
flirt -in betb0.nii.gz -ref betT1.nii.gz -out b02T1.nii.gz -omat b02T1_omat
flirt -in betT1.nii.gz -ref atlas.nii -omat T12atlas_omat
fnirt --in=betT1.nii.gz  --ref=atlas.nii --aff=T12atlas_omat --cout=T12atlas_nonlinear --iout=T12atlas.nii.gz
```

Normalization

A b0 image was first aligned to a native T1 image, and then the native T1 image was normalized to an asymmetric T1 template for 6-12 years from Chinese Paediatric Atlases using linear and nonlinear registration from the FMRIB Software Library (https://fsl.fmrib.ox.ac.uk/fsl).

Normalization template

The 6-12 years from Chinese Paediatric Atlases.
Zhao, T., et al. Unbiased age-specific structural brain atlases for Chinese pediatric population. Neuroimage 189, 55-70 (2019)

Noise and artifact removal

The eddy current distortions and motion artefacts in the dMRI data were corrected by applying an affine alignment of each DWI image to the b0 image.

Volume censoring

Each T1 image was ensured the absence of arachnoid cysts, neuroepithelial cysts, or any other intracranial occupying lesions. Subsequently, five trained raters visual inspected the T1 images for brain damage, missing layers, or evident noise. For dMRI image, images reported as failures by DTIprep were excluded. Additionally, visual inspections by five trained raters were conducted, and images with abnormal volume proportions exceeding 10% were excluded.

Statistical modeling & inference

Model type and settings

Both linear and quadratic models were estimated by a mixed effect model to characterize the intrinsic longitudinal relationship between brain network properties and age. Total brain volume, centre and sex were considered as covariate.

Effect(s) tested

N/A

Specify type of analysis: ☐ Whole brain ☐ ROI-based ☒ Both

Anatomical location(s)

The BNA246 template was used to define brain locations. Briefly, a b0 image was first aligned to a native T1 image, and then the native T1 image was normalized to an asymmetric T1 template for 6-12 years from Chinese Paediatric Atlases using the FMRIB Software Library (https://fsl.fmrib.ox.ac.uk/fsl). Inverse transformation matrices derived from the aforementioned steps were applied to transform the brain atlas of standard space into native space.

Statistic type for inference

(See Eklund et al. 2016)

No voxel-wise or cluster-wise statistic

Correction

Bonferroni correction

Models & analysis

n/a	Involved in the study
☒	☐ Functional and/or effective connectivity
☐	☒ Graph analysis
☐	☒ Multivariate modeling or predictive analysis

Graph analysis

Based on the tractography results, the FA×FN-weighted network of each participant was constructed, where the FA×FN weight was defined as the average FA value of the voxels traversed along the connected fibres between two regions times the number of fibre streamlines (FN) connecting two brain regions. Eight whole-brain properties were calculated according to the constructed network, including global efficiency, local efficiency, shortest path, network strength, clustering coefficient, and small-world parameters (γ, λ and σ). For each brain region, four common nodal properties were calculated: nodal efficiency, nodal local efficiency, nodal degree centrality and nodal betweenness centrality. According to the different categories of two nodes, the existing edges between them were classified into three types: local (nonhub to nonhub), feeder (hub to nonhub) and rich-club (hub to hub).

Multivariate modeling and predictive analysis

Partial least square correlation was performed to mine the weighted linear combinations of gene expression profiles associated with the spatial development slopes of the WM nodal efficiency.
